# Ketogenic Diets Induced Glucose Intolerance and Lipid Accumulation in Mice with Alterations in Gut Microbiota and Metabolites

**DOI:** 10.1128/mBio.03601-20

**Published:** 2021-03-30

**Authors:** Yue Li, Xin Yang, Jing Zhang, Tianyi Jiang, Ziyi Zhang, Zhiyi Wang, Mengxue Gong, Liping Zhao, Chenhong Zhang

**Affiliations:** a State Key Laboratory of Microbial Metabolism, School of Life Sciences and Biotechnology, Shanghai Jiao Tong University, Shanghai, China; b Department of Biochemistry and Microbiology and New Jersey Institute for Food, Nutrition and Health, School of Environmental and Biological Sciences, Rutgers University, New Brunswick, New Jersey, USA; Harvard University

**Keywords:** ketogenic diet, gut microbiota, fecal metabolites, glucose and lipid metabolism

## Abstract

The ketogenic diet (KD), which can induce changes in gut microbiota, has shown benefits for epilepsy and several neurodegenerative diseases. However, the effects of a KD on glucose and lipid metabolism remain inconclusive. Using two formulas of ketogenic diets (KDR with 89.5% fat and KDH with 91.3% fat), which are commonly used in mouse trials, we found that KDR but not KDH induced insulin resistance and damaged glucose homeostasis, while KDH induced more fat accumulation in mice. Further study showed that KD impacted glucose metabolism, which was related to the sources of fat, while both the sources and proportions of fat affected lipid metabolism. And the KD widely used in human studies still induced insulin resistance and fat accumulation in mice. Moreover, KDs changed the gut microbiota and metabolites in mice, and the sources and proportions of fat in the diets respectively changed the abundance of specific bacteria and metabolites which were correlated with parameters related to glucose intolerance and lipid accumulation. Overall, our study demonstrated that the metabolic disorders induced by KDs are closely related to the source and proportion of fat in the diet, which may be associated with the changes of the gut microbiota and metabolites.

## Abstract (precis)

The ketogenic diet with extremely high fat and very low carbohydrate levels is very popular in society today. Although it has beneficial effects on epilepsy and neurodegenerative diseases, how ketogenic diets impact host glucose and lipid metabolism and gut microbiota still needs further investigation.

## INTRODUCTION

Ketogenic diet (KD) refers to an extremely high-fat, very low-carbohydrate diet, which has shown its value in treating epilepsy and its increasing application to other diseases, such as autism spectrum disorder, Alzheimer’s disease, and metabolic syndrome (1, 2). However, reports about the effects of a KD on glucose and lipid metabolism in rodents and humans remain inconclusive. Previous clinical trials showed the effectiveness of weight loss and improvement in glucose tolerance and serum lipids in overweight/obese subjects (3–6). However, a 3-day KD increased postprandial plasma glucose in healthy men (7). Although it decreased triglycerides, a KD increased cholesterol and inflammatory markers in overweight/obese subjects after 4 weeks of dietary intervention (8). In a rodent trial, a KD reduced circulating glucose and lipids in both normal-weight and ob/ob mouse models (9) and improved glucose tolerance in diet-induced obese mice (10). Nevertheless, several studies found that a KD has negative effects, including hepatic steatosis, insulin resistance, and glucose intolerance (11–17), and a recent study showed that 2 to 4 months of KD feeding depleted adipose-resident γδ T cells, which could restrain inflammation, resulting in impaired glucose homeostasis (18). Although the diets mentioned above are all called a “ketogenic diet,” the differences in ingredients and dietary composition even in more controlled and standardized animal studies still exist. Therefore, clearly exploring the impact of a KD on glucose and lipid metabolism is needed.

The gut microbiota is critically influenced by diet and has been considered a key mediator linking diet and host physiology (19). Previous work found that a KD altered the structure of gut microbiota, and specific KD-associated bacteria could protect against seizures in mouse models of intractable epilepsy, which was further confirmed because the protective effects disappeared when mice were treated with antibiotics or reared germfree (20). Furthermore, a recent study showed that a KD decreased the abundance of *Bifidobacterium*, and KD-associated gut microbiota reduced the levels of intestinal proinflammatory Th17 cells (21). Therefore, whether the gut microbiota altered by a KD affects host glucose and lipid metabolism became an interesting question, as did whether microbe-associated metabolites also play a role.

Here, to evaluate the effects of KD on host glucose and lipid metabolism, gut microbiota, and microbe-associated metabolites, two kinds of ketogenic diets commonly used in mouse trials, type 1 (KDR) and type 2 (KDH), were investigated in mice. We found that KDR but not KDH induced insulin resistance and damage to glucose homeostasis, while KDH induced more fat accumulation in mice, which was associated with their distinct effects on the gut microbiota and metabolite profiles. Moreover, we showed that the differences in metabolic phenotypes in mice induced by these two kinds of KD may be due to the sources and proportions of fat in the diet.

## RESULTS

### Ketogenic diets induced glucose intolerance and lipid accumulation in mice.

To observe how two kinds of ketogenic diets impacted physiological and metabolic consequences in mice, 8-week-old male C57BL/6J mice were randomly assigned into four groups: (i) fed ketogenic diet type 1 (KDR, caloric ratio of 89.5% fat, 0.1% carbohydrate, and 10.4% protein), (ii) fed control normal chow of KDR (NCR, caloric ratio of 10% fat, 70% carbohydrate, and 20% protein), (iii) fed ketogenic diet type 2 (KDH, caloric ratio of 91.3% fat, 1% carbohydrate, and 7.7% protein), and (iv) fed control normal chow of KDH (NCH, caloric ratio of 15.5% fat, 64.5% carbohydrate, and 20% protein). Details of these diets are shown in Table S1 in the supplemental material. β-Hydroxybutyrate concentrations in blood significantly increased in the mice fed the two kinds of KD, and there was no difference between the KDR and KDH groups (Fig. S1a).

10.1128/mBio.03601-20.1 FIG S1 The effects of KDs on β-hydroxybutyrate, weight, and intestinal barrier in mice. (a) Blood ketone (β-hydroxybutyrate) levels. (b) Curves of blood glucose levels during OGTT and AUC after 2 weeks of dietary intervention. (c) The body weight of each group after 5 weeks of dietary intervention. (d) Relative expression of *Occludin*, *Muc2*, *Zo-1*, *Muc1*, and *Muc3* in the colon. Differences are analyzed using *t* test. (e) Representative immunohistochemistry-stained (brown) sections for ZO-1 of ileum (scale bar = 100 μm) and calculated average integrated optical density (IOD). (f) Serum level of LBP. Data are presented as the mean ± SEM and analyzed using one-way ANOVA, followed by a Tukey *post hoc* test, except for panel d. *, *P* < 0.05; **, *P* < 0.01; ***, *P* < 0.001. *n* = 8 to 9 for both groups for all analyses. Download FIG S1, PDF file, 2.9 MB. Copyright © 2021 Li et al. 2021 Li et al. https://creativecommons.org/licenses/by/4.0/ This content is distributed under the terms of the Creative Commons Attribution 4.0 International license.

10.1128/mBio.03601-20.8 TABLE S1 The formulas of diets in animal trials. Download Table S1, DOCX file, 0.01 MB. Copyright © 2021 Li et al. 2021 Li et al. https://creativecommons.org/licenses/by/4.0/ This content is distributed under the terms of the Creative Commons Attribution 4.0 International license.

**FIG 1:**
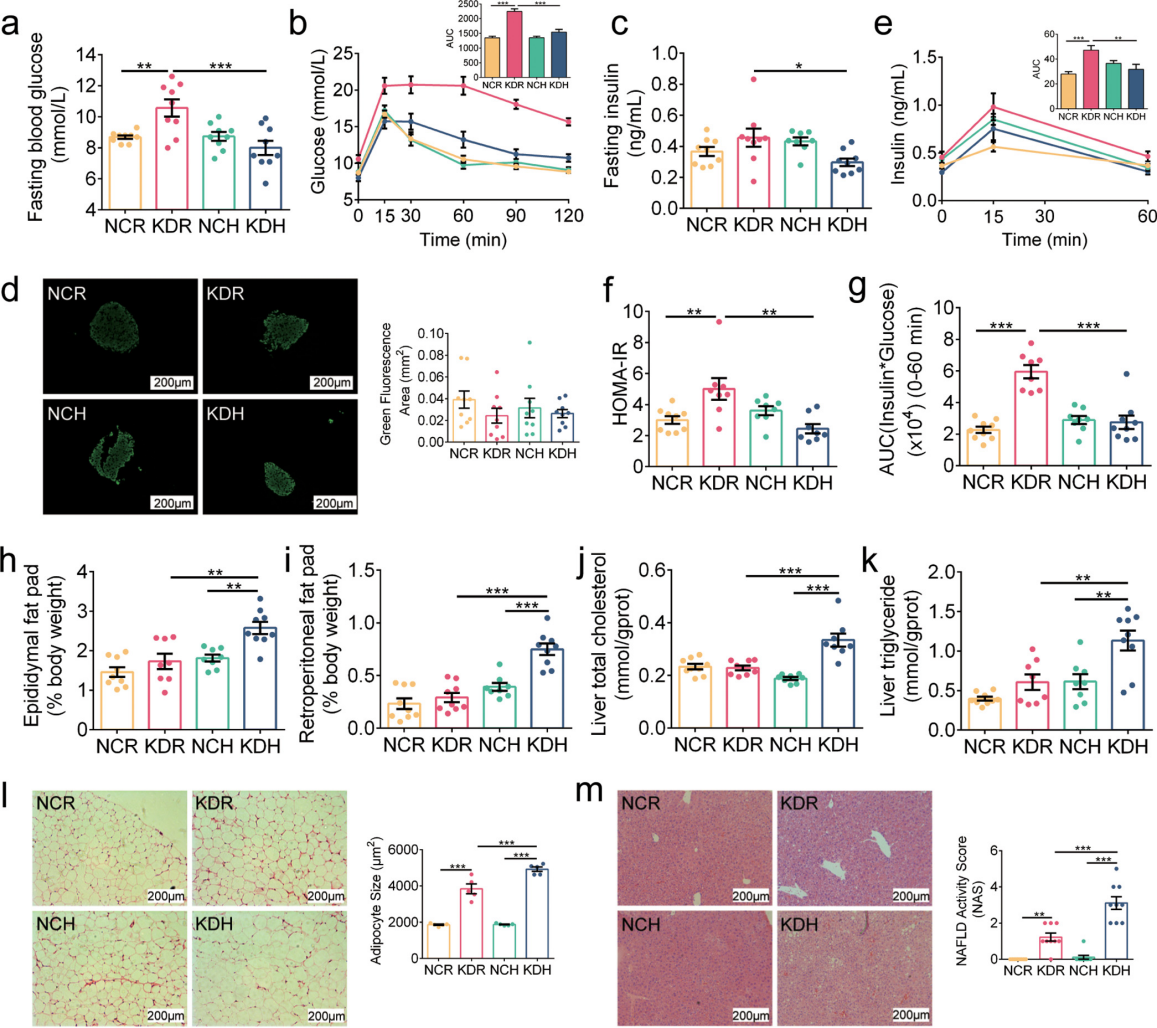
KD induced glucose intolerance and lipid accumulation in mice. (a) Fasting blood glucose. (b) Curves of blood glucose levels during oral glucose tolerance test (OGTT) and areas under the curve (AUC). (c) Fasting serum insulin. (d) Representative insulin immunofluorescence-stained (green) histological sections of pancreas (scale bar = 200 μm) and calculated mean area of islet. (e) Serum insulin levels during OGTT and AUC. (f) Homeostasis model assessment of insulin resistance (HOMA-IR). (g) Insulin resistance index (the product of the 0- to 60-min AUC of blood glucose and that of serum insulin in OGTT). (h and i) Epididymal and retroperitoneal fat mass (% body weight), respectively. (j and k) Concentrations of total cholesterol and triglyceride in liver, respectively. (l) Representative H&E-stained histological sections of epididymal adipose tissue (eAT) (scale bar = 200 μm) and calculated mean cell area of adipocytes (*n* = 5 per group). (m) Representative H&E-stained histological sections of liver (scale bar = 200 μm) and calculated histologic score (NAFLD activity score). Data are presented as the mean ± SEM and analyzed using one-way ANOVA, followed by a Tukey *post hoc* test. *, *P* < 0.05; **, *P < *0.01; ***, *P* < 0.001. *n* = 8 to 9 for both groups for all analyses except *n* = 5 for panel l.

First, we observed how these two kinds of KD affected glucose metabolism. We did oral glucose tolerance test (OGTT) after 2 and 5 weeks of dietary intervention and found that the KDR mice showed significantly higher fasting blood glucose levels than the NCR mice, but there was no significant difference between the mice in the KDH and NCH groups (Fig. 1a). Moreover, we found that the KDR mice but not KDH mice had slower glucose clearance than their control group during the OGTT (Fig. 1b and Fig. S1b). Although the secretion of insulin was similar among the four groups of mice, which was confirmed by fasting blood insulin levels and immunofluorescence staining for insulin in the pancreas (Fig. 1c and d), the homeostatic model assessment for insulin resistance (HOMA-IR) index and insulin resistance index were significantly increased in the KDR mice (Fig. 1e to g). These results suggested that KDR but not KDH induced insulin resistance and damage to glucose homeostasis in mice.

Next, we observed how these two kinds of KD impacted lipid metabolism. After 5 weeks of dietary intervention, the KDH mice had lower body weights (Fig. S1c) and higher weights of epididymal and retroperitoneal fat mass (Fig. 1h and i) than the NCH mice. Furthermore, the KDH mice also exhibited excessive lipid accumulation in the liver with increased concentrations of liver total cholesterol and triglyceride compared to the NCH mice (Fig. 1j and k). However, there was no significant difference in the above parameters between the mice in the KDR and NCR groups. Both the adipocyte size and nonalcoholic fatty liver disease (NAFLD) activity score significantly increased in the mice fed the two kinds of KD compared to their respective control groups, which were estimated by hematoxylin and eosin (H&E)-stained epididymal fat and liver, and the KDH mice had significantly higher values than the KDR mice (Fig. 1l and m). These results suggested that KDH induced more fat accumulation in mice than KDR.

Previous studies have shown that metabolic diseases, including type 2 diabetes (T2D) and nonalcoholic fatty liver disease (NAFLD), which disrupt glucose and lipid metabolism, are characterized by an impaired and defective intestinal barrier (22). To determine whether intestinal permeability was impacted by two kinds of KD, we measured the expression of *Zo-1*, *Occludin*, *Muc1*, *Muc2*, and *Muc3* in the colon. The KDR and KDH mice showed significantly lower expression of *Occludin* and *Muc2* than their control groups, implying that the intestinal barrier was damaged in the mice fed with the two kinds of KD (Fig. S1d), which was further confirmed by immunohistochemistry staining for ZO-1 in the ileum (Fig. S1e). Moreover, we found that the serum level of lipopolysaccharide (LPS)-binding protein (LBP) was significantly enhanced in the mice fed with the two kinds of KD, implying increased intestinal permeability to LPS, and there was no difference between the KDR and KDH mice (Fig. S1f). These results suggested that both KDR and KDH impaired intestinal barrier function in mice.

### Two kinds of ketogenic diets molded disparate gut microbiota in mice.

**FIG 2:**
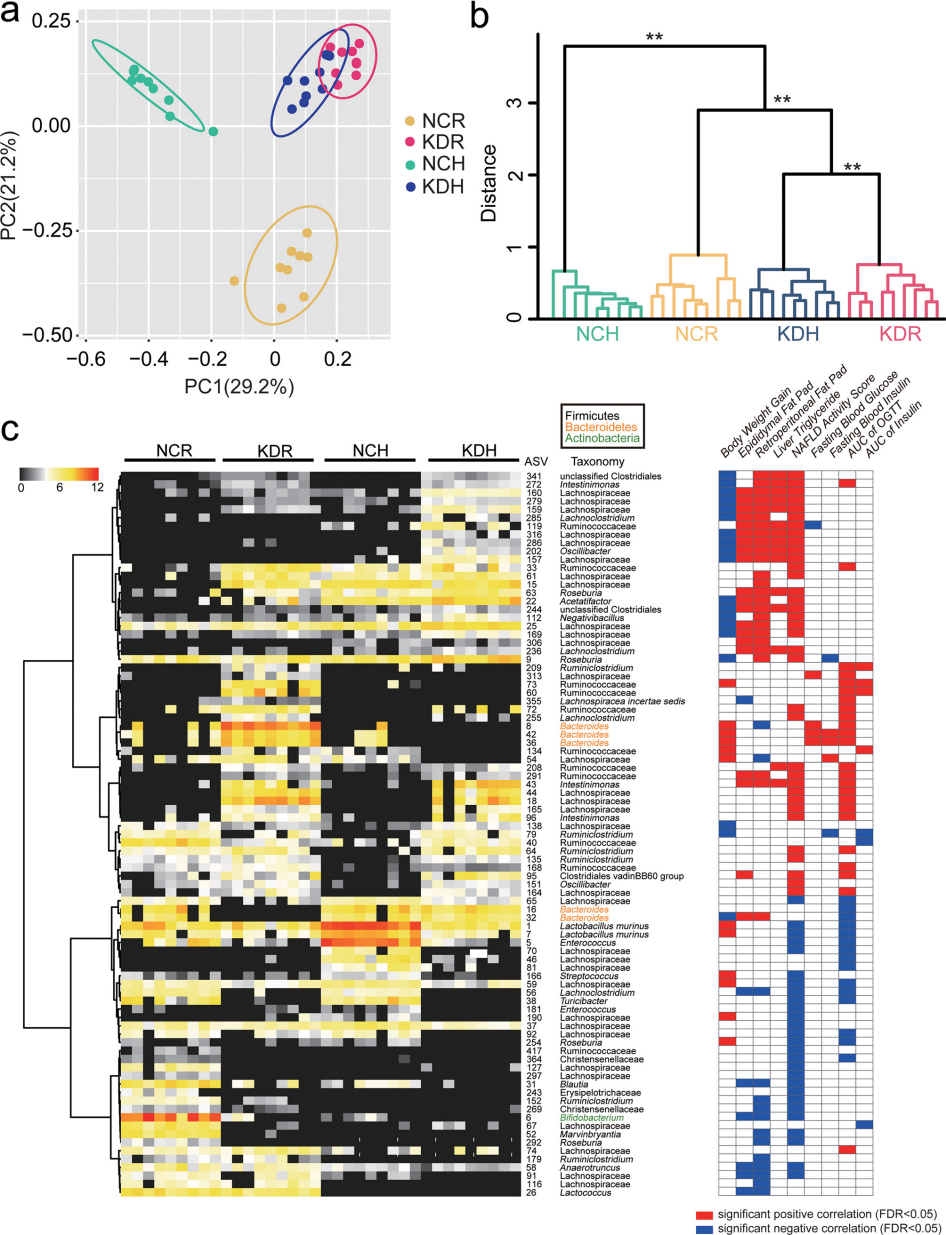
Two kinds of KD molded disparate gut microbiota in mice. (a) Principal-coordinate analysis (PCoA) plot of gut microbiota based on the Bray-Curtis distance. (b) Clustering of the gut microbiota between different groups calculated with permutational multivariate analysis of variances (PERMANOVA; 9,999 permutations) using Bray-Curtis distances. **, *P* < 0.01. (c) Left, the heatmap represents the normalized and log_2_-transformed relative abundance of the 87 ASVs in each sample. These ASVs were clustered by the ward.D method. Right, Spearman correlations between the relative abundance of 87 ASVs and the host parameters related to glucose and lipid metabolism. *P* values of correlations were adjusted by false-discovery rate (FDR). Only where FDR was <0.05, red and blue denote significant positive and negative correlation, respectively.

By quantitative real-time PCR (qPCR) targeting the 16S rRNA gene, we found that the total bacterial loads in feces were similar among the four groups of mice (Fig. S2a). To analyze whether the structure of gut microbiota was modulated by these two kinds of KD, we sequenced the 16S rRNA gene V3-V4 region of fecal samples collected at the 5th week. After 5 weeks of intervention, the richness and diversity of gut microbiota, which were reflected by the numbers of observed amplicon sequence variants (ASVs) and the Shannon index, were significantly increased only in the KDH mice but not in the KDR mice compared to their control groups (Fig. S2b). Principal-coordinate analysis (PCoA) and permutational multivariate analysis of variance (PERMANOVA) based on Bray-Curtis distances showed clear separations of gut microbiota structure between the mice in the KDR and NCR groups, as well as the KDH and NCH groups (*P* < 0.01 with PERMANOVA, 9,999 permutations). Although the gut microbiota structures were more similar in the mice fed the two kinds of KD than in mice fed the two control diets, there was still a significant difference between the KDR and KDH mice (Fig. 2a and b).

10.1128/mBio.03601-20.2 FIG S2 Total fecal bacterial concentrations, observed ASVs, and Shannon index of each group after 5 weeks of dietary intervention. For panel a, data are presented as the mean ± SEM and analyzed using one-way ANOVA, followed by a Tukey *post hoc* test. For panel b, data are analyzed using Kruskal-Wallis test. *, *P* < 0.05. *n* = 9 for both groups for all analyses. Download FIG S2, PDF file, 0.9 MB. Copyright © 2021 Li et al. 2021 Li et al. https://creativecommons.org/licenses/by/4.0/ This content is distributed under the terms of the Creative Commons Attribution 4.0 International license.

We constructed sparse partial least-squares discriminant analysis (sPLS-DA) models to identify members of the gut microbiota responding to these two kinds of KD intervention. We found that 44 ASVs were shifted in the KDR mice compared to the NCR mice, while 40 ASVs showed differences between the KDH and NCH mice (Fig. S3a and b). Notably, only 16 ASVs showed the same changes in both the KDR and KDH mice. Of these, the abundances of ASVs, including *Ruminococcaceae* (ASV208, ASV291), *Intestinimonas* (ASV43, ASV96, ASV272), and *Lachnospiraceae* (ASV44, ASV18, ASV159, ASV164, ASV165), were significantly enriched by both types of KD, and all these ASVs positively correlated with parameters related to glucose intolerance and lipid accumulation. Furthermore, 6 ASVs, including *Enterococcus* (ASV5), *Blautia* (ASV31), *Turicibacter* (ASV38), *Lachnoclostridium* (ASV56), and *Lachnospiraceae* (ASV59, ASV65), were significantly decreased by both KDs, and all these ASVs negatively correlated with parameters related to glucose intolerance and lipid accumulation. Next, we compared the gut microbiota in mice with the two types of KD and identified 38 significantly different ASVs (Fig. S3c). Among these 38 ASVs, *Bacteroides* (ASV8, ASV36, ASV42), *Lachnospiraceae* (ASV313), and *Ruminococcaceae* (ASV60, ASV73) were significantly enriched in the KDR mice and only positively correlated with parameters related to glucose intolerance. Moreover, *Roseburia* (ASV9, ASV63), *Negativibacillus* (ASV112), *Acetatifactor* (ASV22), *Oscillibacter* (ASV202), *Lachnoclostridium* (ASV285), and *Lachnospiraceae* (ASV25, ASV157, ASV159, ASV160, ASV169, ASV279, ASV286, ASV316) were significantly increased in the KDH mice and only positively correlated with parameters related to lipid accumulation (Fig. 2c). Taken together, the results suggested that two types of KD introduced different changes in the gut microbiota.

10.1128/mBio.03601-20.3 FIG S3 ASVs differentiating gut microbiota structure between the mice of different groups. The sPLS-DA plot of gut microbiota, the classification error rate of one component across the leave-one-out cross-validation for maximum distance, and the heatmap of selected ASVs. (a) NCR versus KDR. (b) NCH versus KDH. (c) KDR versus KDH. Download FIG S3, PDF file, 1.1 MB. Copyright © 2021 Li et al. 2021 Li et al. https://creativecommons.org/licenses/by/4.0/ This content is distributed under the terms of the Creative Commons Attribution 4.0 International license.

### Changes in the metabolome in mice fed two kinds of ketogenic diets.

**FIG 3:**
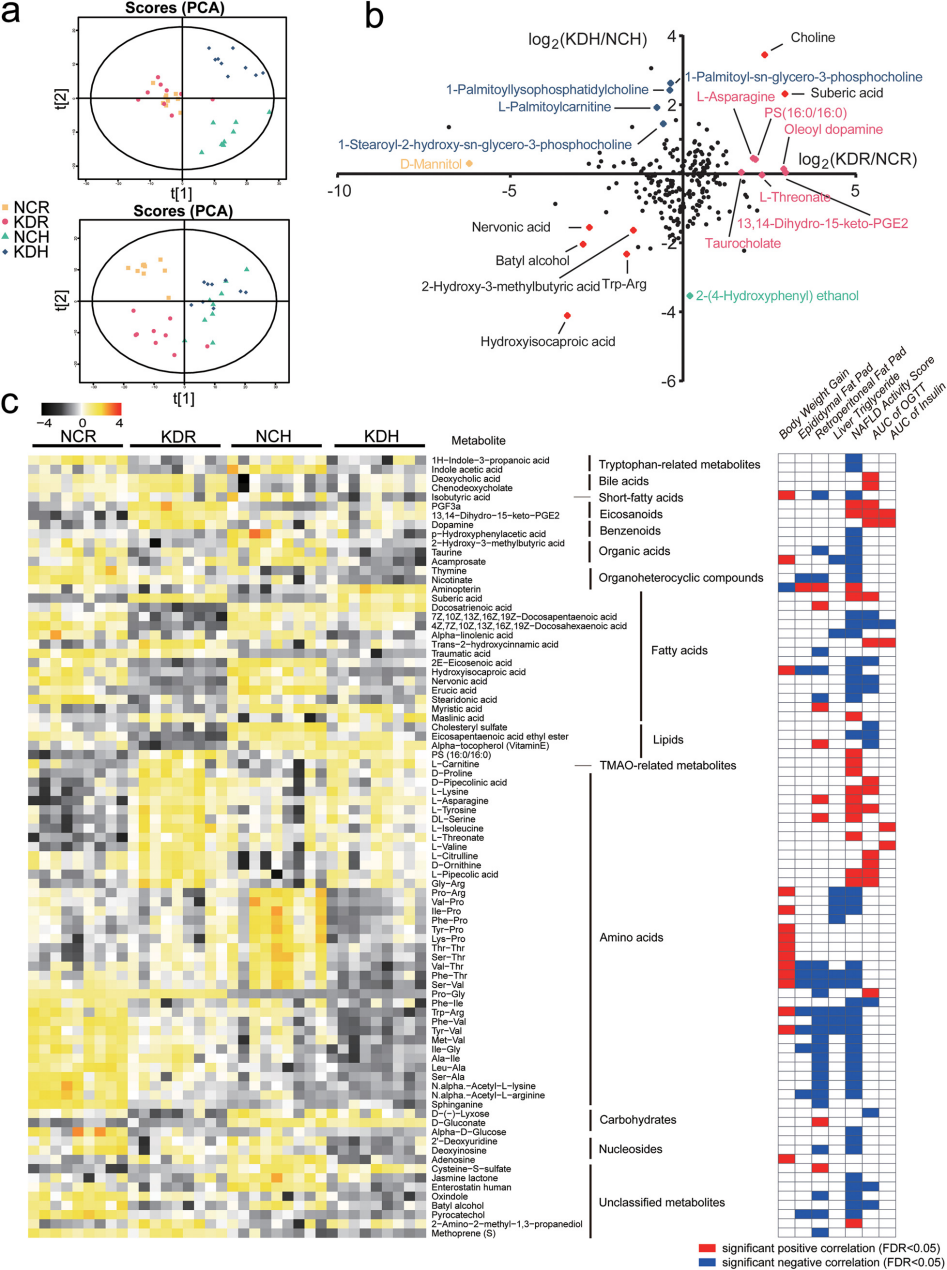
Changes of the metabolome in mice fed with two kinds of KD. (a) Principal-component analysis (PCA) plots of metabolic profiles. Top, ESI^+^; bottom, ESI−. (b) The fold ratio (log_2_-transformed) of the abundance of 226 identified metabolites in the KDR to NCR mice, and the KDH toNCH mice. The red spots represent the metabolites that enriched or decreased in both the KDR and KDH groups, and the rose red, orange, blue, and mint green spots represent the metabolites that enriched in the KDR, NCR, KDH, and NCH mice, respectively. (c) Left, the heatmap represents the abundance of the 85 metabolites in each sample. Right, Spearman correlations between the abundance of 85 metabolites and the host parameters related to glucose and lipid metabolism. *P* values of correlations were adjusted by FDR. Only where FDR was <0.05, red and blue denote significant positive and negative correlation, respectively.

To identify how these two kinds of KD affected metabolites of the gut microbiota in mice, the untargeted metabolome of the cecum content was analyzed by liquid chromatography-mass spectrometry (LC-MS), and principal-component analysis (PCA) showed that the metabolome profiles of the gut microbiota in the KDR and KDH mice were significantly different from those of their control groups. Notably, the metabolite profiles of the KDR and KDH mice clearly segregated in the first principal component (Fig. 3a).

From orthogonal projection to latent structure-discriminant analysis (OPLS-DA) models (Fig. S4), we identified differential metabolic features (variable importance in projection [VIP] > 1, *P* < 0.05) that included 127 metabolites between the KDR and NCR mice, 125 metabolites between the KDH and NCH mice, and 117 metabolites between the KDH and KDR mice (Data Set S1). A total of 156 of these metabolites were significantly correlated with host parameters related to glucose and lipid metabolism (Data Set S2). In addition, 85 of these 156 metabolites were significantly correlated with gut microbial variations (*R*^2^ > 0.09, *P* < 0.05 of PERMANOVA) (Data Set S2). Among these 85 metabolites, suberic acid was significantly enhanced in both the KDR and KDH mice, and it positively correlated with parameters related to glucose intolerance and lipid accumulation. Moreover, nervonic acid and batyl alcohol, which were significantly decreased by both KDs, were negatively correlated with parameters related to glucose intolerance and lipid accumulation. Besides, two bile acids, deoxycholic acid (DCA) and chenodeoxycholate (CDCA), were positively correlated with glucose intolerance. They were increased in both the KDR and KDH mice, but the KDR mice had significantly higher levels than the KDH mice. 1H-indole-3-propanoic acid (IPA) and indole acetic acid (IAA), which are the products of the bacteria metabolizing tryptophan, were negatively correlated with lipid accumulation. They were decreased in the KDR and KDH mice, respectively. Furthermore, trans-2-hydroxycinnamic acid, which increased only in the KDR mice, was positively correlated with glucose intolerance, while isobutyric acid, which decreased in the KDH mice, was negatively correlated with lipid accumulation (Fig. 3b and c). Taken together, these results suggested that KD intervention changed the metabolites in the gut, and two types of KD also had different influences.

10.1128/mBio.03601-20.4 FIG S4 The OPLS-DA score plots of metabolic profiles in cecum content of mice. (a) NCR versus KDR. (b) NCH versus KDH. (c) KDR versus KDH. Download FIG S4, PDF file, 0.9 MB. Copyright © 2021 Li et al. 2021 Li et al. https://creativecommons.org/licenses/by/4.0/ This content is distributed under the terms of the Creative Commons Attribution 4.0 International license.

10.1128/mBio.03601-20.9 DATA SET S1 The differentiation of metabolites between NCR and KDR, NCH and KDH, or KDR and KDH mice. Download Data Set S1, XLSX file, 0.03 MB. Copyright © 2021 Li et al. 2021 Li et al. https://creativecommons.org/licenses/by/4.0/ This content is distributed under the terms of the Creative Commons Attribution 4.0 International license.

10.1128/mBio.03601-20.10 DATA SET S2 One hundred fifty-six metabolites were significantly correlated with at least one host parameter related to glucose and lipid metabolism, and 85 of these 156 metabolites were significantly correlated with gut microbial variations. Download Data Set S2, XLSX file, 0.04 MB. Copyright © 2021 Li et al. 2021 Li et al. https://creativecommons.org/licenses/by/4.0/ This content is distributed under the terms of the Creative Commons Attribution 4.0 International license.

### The sources and proportions of fat in the ketogenic diet affected the metabolic phenotypes in mice.

**FIG 4:**
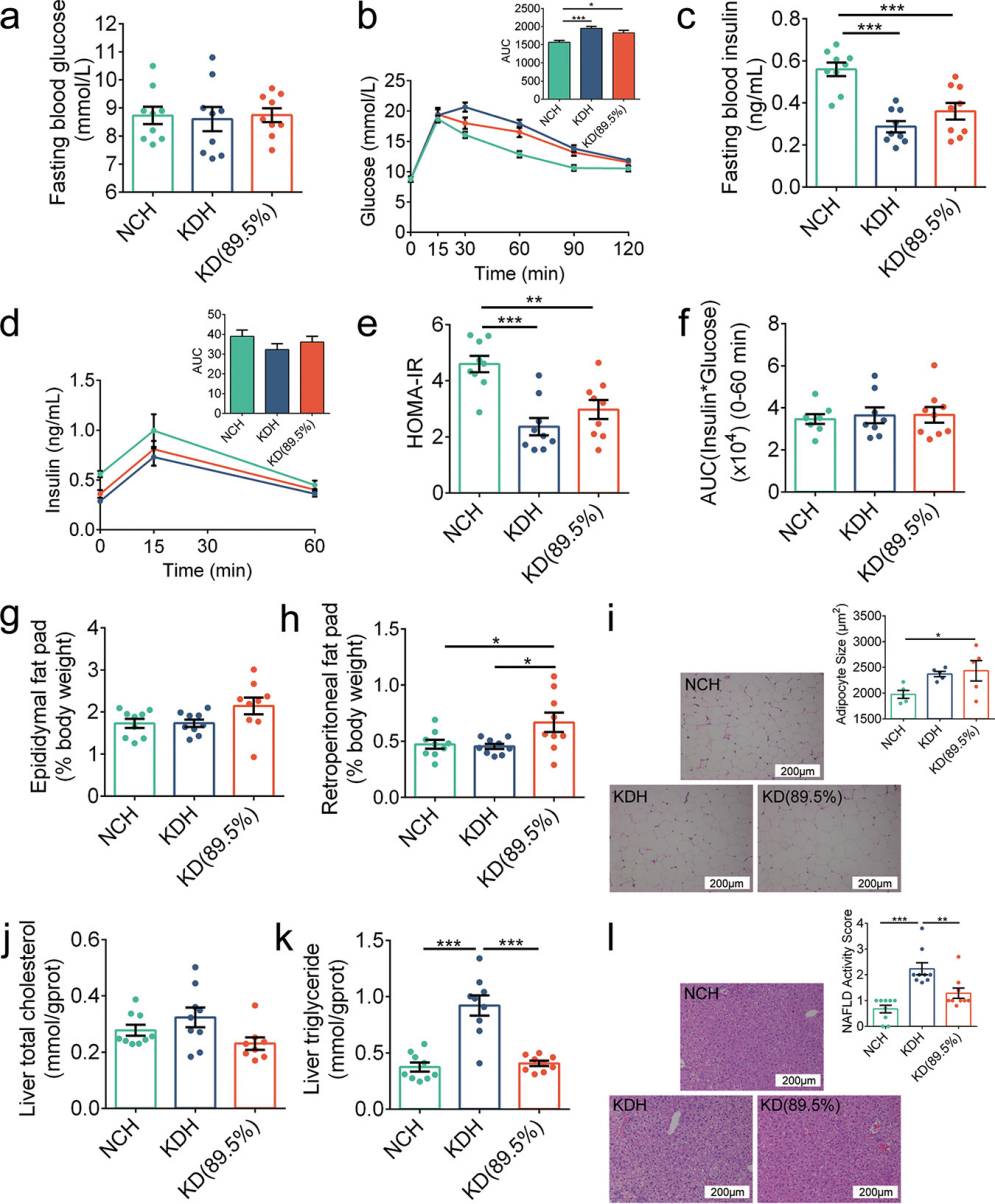
The sources and proportions of fat in KDs affected the metabolic phenotypes in mice. (a) Fasting blood glucose. (b) Curves of blood glucose levels during OGTT and AUC. (c) Fasting serum insulin. (d) Serum insulin levels during OGTT and AUC. (e) HOMA-IR. (f) Insulin resistance index. (g and h) Epididymal and retroperitoneal fat mass (% body weight), respectively. (i) Representative H&E-stained histological sections of eAT (scale bar = 200 μm) and calculated mean cell area of adipocytes (*n* = 5 per group). (j and k) Concentrations of total cholesterol and triglyceride in liver, respectively. (l) Representative H&E-stained histological sections of liver (scale bar = 200 μm) and NAFLD activity score. Data are presented as the mean ± SEM and analyzed using one-way ANOVA, followed by a Tukey *post hoc* test. *, *P* < 0.05; **, *P* < 0.01; ***, *P* < 0.001. *n* = 8 to 9 for both groups for all analyses, except *n* = 5 for panel i.

The two kinds of KD induced different damage to metabolic phenotypes in mice, which was associated with gut microbiota and metabolite changes, although the components of macronutrients were virtually identical in KDR and KDH. Then, we customized a ketogenic diet called KD(89.5%) with the same proportions of fat as KDR and the same sources as KDH in nutrients to evaluate the influences of sources and proportions of fat in the diets on the metabolic phenotypes in mice. We obtained comparable results for parameters related to glucose and lipid metabolism for the mice fed KDH and NCH to those in the above-mentioned trial. Unlike the mice in the KDR group, which had dramatic damage to glucose metabolism, the KD(89.5%) mice had fasting blood glucose levels, glucose clearance, and insulin resistance indices similar to the KDH mice (Fig. 4a to f). Furthermore, the KD(89.5%) mice exhibited an increase in epididymal and retroperitoneal fat mass compared to the NCH mice (Fig. 4g to i), which was not observed between the mice in the KDR and NCR groups. Interestingly, the KD(89.5%) mice did not develop excessive lipid accumulation in the liver like the KDH mice (Fig. 4j to l), indicating that only KDH, which contained 91.3% fat, induced lipid accumulation in the livers of mice. Taken together, these results suggested that the sources but not proportions of fat in the KD induced differences in metabolic phenotypes related to glucose metabolism, while both the sources and proportions induced differences in metabolic phenotypes related to lipid metabolism.

Based on the above results, we concluded that the sources of fat in ketogenic diets had important and significantly different effects on metabolic phenotypes in mice. Therefore, we quantified the concentrations of fatty acids in the three different ketogenic diets [KDR, KDH, and KD(89.5%)] and found that trans-fatty acids (TFAs) were dramatically increased in KDR, which was confirmed by the concentrations of trans-6-octadecenoic acid, trans-9-octadecenoic acid, and trans-11-octadecenoic acid, and there was no significant difference between KDH and KD(89.5%) (Fig. S5). Combining the results that KDR but not KDH induced insulin resistance and damaged glucose homeostasis in mice, we speculated that the trans-fatty acids (TFAs) in KDR may be an important suspect.

10.1128/mBio.03601-20.5 FIG S5 The concentrations of trans-6,9,11-octadecenoic acid in three different ketogenic diets [KDR, KDH, and KD(89.5%)]. Data are presented as the mean ± SEM and analyzed using one-way ANOVA, followed by a Tukey *post hoc* test. ***, *P* < 0.001. Download FIG S5, PDF file, 0.9 MB. Copyright © 2021 Li et al. 2021 Li et al. https://creativecommons.org/licenses/by/4.0/ This content is distributed under the terms of the Creative Commons Attribution 4.0 International license.

### The gut bacteria and metabolites affected by the sources and proportions of fat in ketogenic diets.

**FIG 5:**
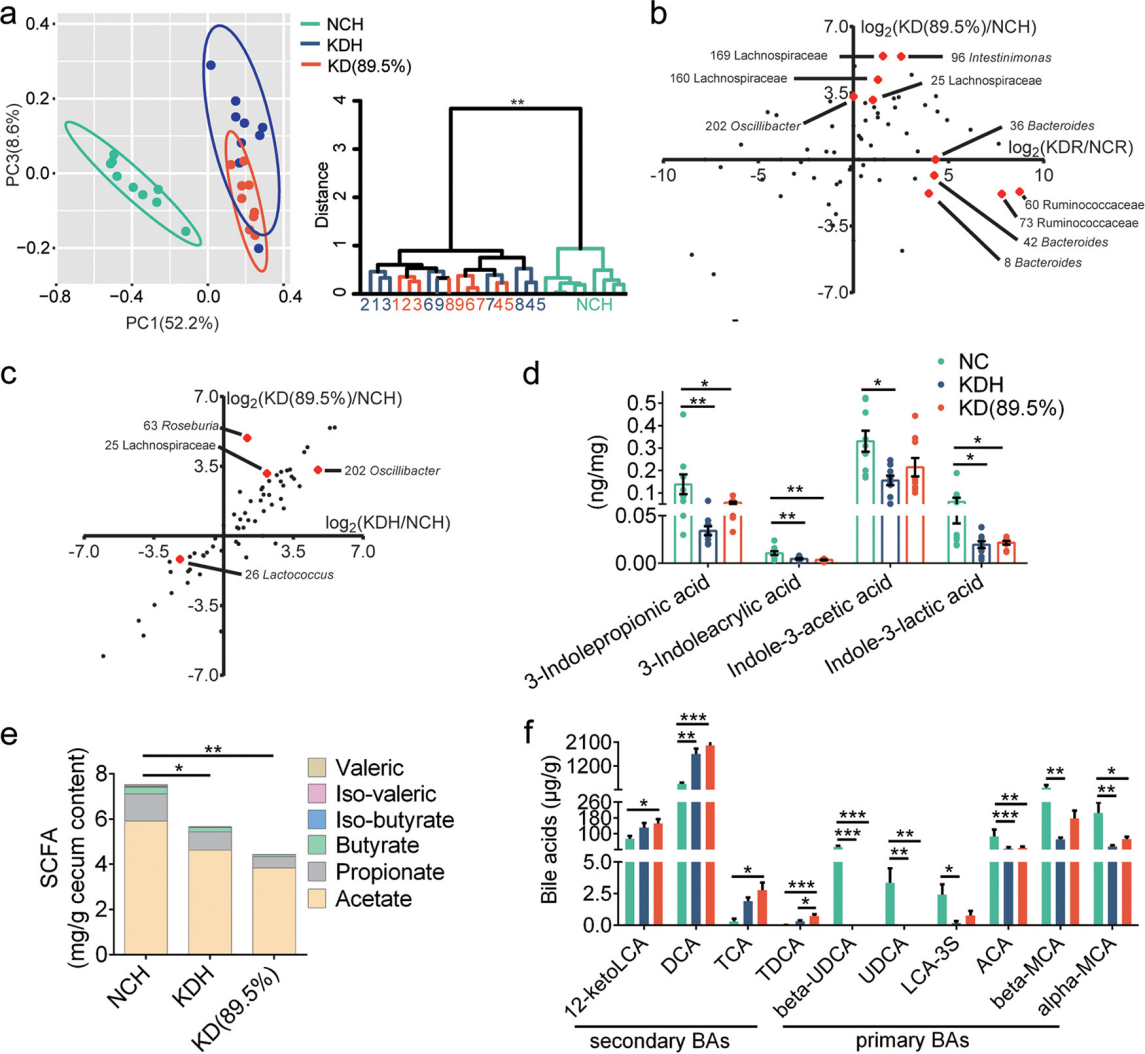
The gut microbiota and metabolites affected by the sources and proportions of fat in KDs. (a) Left, PCoA plot of gut microbiota based on the Bray-Curtis distance. Right, clustering of the gut microbiota between different groups calculated with PERMANOVA (9,999 permutations) using Bray-Curtis distances. **, *P* < 0.01. (b) The fold ratio (log_2_ transformed) of the relative abundance of 87 ASVs in the KD(89.5%) to NCH mice, and KDR to NCR mice. The red spots represent the ASVs significantly different between two KD groups fed KDs with the same proportion but different sources of fat. (c) The fold ratio (log_2_ transformed) of the relative abundance of 87 ASVs in the KD(89.5%) to NCH mice, and KDH to NCH mice. The red spots represent the ASVs significantly different between two KD groups fed KDs with the same source but different proportions of fat. (d) The concentrations of tryptophan metabolites in fecal samples of mice. (e) The concentrations of SCFAs in cecum content of mice (*n* = 5 to 6 per group). (f) The concentrations of bile acids (BAs) in cecum content of mice. For panels d to f, data are presented as the mean ± SEM and analyzed using one-way ANOVA followed by a Tukey *post hoc* test. *, *P* < 0.05; **, *P* < 0.01; ***, *P* < 0.001. *n* = 8 to 9 for both groups for all analyses, except *n* = 5 to 6 for panel e.

By 16S rRNA gene sequencing, we found that there were still significant differences in the gut microbiota between the mice in the KDH and KD(89.5%) groups (*P* < 0.05 with PERMANOVA, 9,999 permutations) (Fig. 5a). Next, we observed how the abundances of 87 ASVs affected by two kinds of KD in the above-mentioned trial changed. Of the mice fed these two ketogenic diets with the same proportion but different sources of fat, the KD(89.5%) mice had an almost negligible abundance of *Bacteroides* (ASV8, ASV36, ASV42) and *Ruminococcaceae* (ASV60, ASV73), similar to the NCH mice but unlike the KDR mice, which showed significant enrichment in these microbes. However, the KD(89.5%) mice exhibited a significant increase in the abundance of *Lachnospiraceae* (ASV25, ASV160, ASV169), *Intestinimonas* (ASV96), and *Oscillibacter* (ASV202), and there was no significant difference between the KDR and NCR mice (Fig. 5b and Fig. S6a). In addition, for these two ketogenic diets with the same source but different proportions of fat, although the abundances of most ASVs were similar between the KDH and KD(89.5%) mice, differences still existed. KD(89.5%) significantly increased the abundance of ASVs, including ASV25 (*Lachnospiraceae*), ASV26 (*Lactococcus*), and ASV63 (*Roseburia*), and decreased the abundance of ASV202 (*Oscillibacter*) compared to the KDH mice (Fig. 5c and Fig. S6b). Taken together, we identified 10 ASVs and 4 ASVs that responded to the sources and proportions of fat in ketogenic diets, respectively. Thus, these 14 ASVs were considered key members that were affected by the sources and proportions of fat in the two kinds of KD.

10.1128/mBio.03601-20.6 FIG S6 The gut microbiota affected by the sources and proportions of fat in KDs. (a) The heatmap represents the normalized and log_2_-transformed relative abundance of the 83 ASVs (the abundance of 4 ASVs was zero) in mice. The mice of these two KD groups were fed KDs with the same proportion but different sources of fat. The ASVs were clustered by the ward.D method. (b) The heatmap represents the normalized and log_2_-transformed relative abundance of the 63 ASVs (the abundance of 24 ASVs was zero) in mice. The mice of these two KD groups were fed KDs with the same source but different proportions of fat. The ASVs were clustered by the ward.D method. Download FIG S6, PDF file, 1.6 MB. Copyright © 2021 Li et al. 2021 Li et al. https://creativecommons.org/licenses/by/4.0/ This content is distributed under the terms of the Creative Commons Attribution 4.0 International license.

From untargeted metabolome data, we showed that KDs affected many of the gut microbiota-related metabolites, such as short-chain fatty acids (SCFAs), tryptophan metabolites, and bile acids (BAs). Thus, we quantified the concentrations of 4 main tryptophan metabolites, 6 SCFAs, and 40 BAs with LC-MS or gas chromatography (GC)-MS. Consistent with the results in untargeted metabolome, we also observed decreased tryptophan metabolites in both the KDH and KD(89.5%) mice (Fig. 5d). Moreover, we found that the concentrations of SCFAs significantly decreased in the KDH and KD(89.5%) mice, and there was no significant difference between the mice of these two groups. This suggested that decreased SCFAs may be related to lack of carbohydrates in the KD (Fig. 5e). Furthermore, we observed the concentrations of 10 BAs were significantly different in the mice of different groups. Among them, DCA, which belongs to secondary BAs, was significantly increased in both the KDH and KD(89.5%) mice. Notably, the KDR mice had significantly higher DCA and CDCA levels than the KDH mice in untargeted metabolome, but there was no significant difference between the mice in the KD(89.5%) and KDH groups, suggesting that the differences of DCA and CDCA levels in the KDR and KDH mice may be due to different fat sources in the KD (Fig. 5f).

### The ketogenic diet used in human studies still impaired metabolic health, gut microbiota, and metabolites in mice.

**FIG 6:**
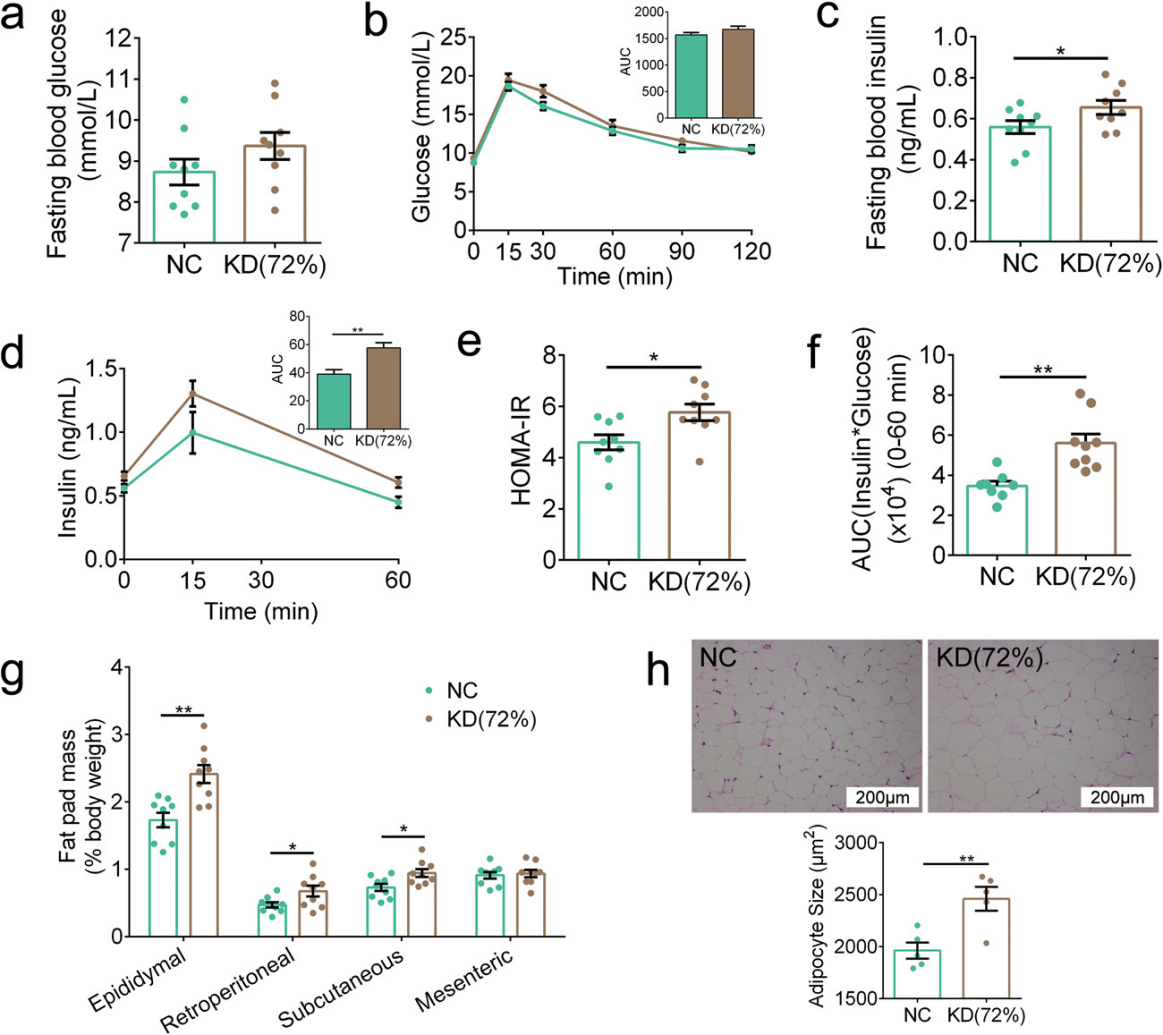
KD(72%) impaired metabolic health in mice. (a) Fasting blood glucose. (b) Curves of blood glucose levels during OGTT and AUC. (c) Fasting serum insulin. (d) Serum insulin levels during OGTT and AUC. (e) HOMA-IR. (f) Insulin resistance index. (g) Fat pad mass (% body weight). (h) Representative H&E-stained histological sections of eAT (scale bar = 200 μm) and calculated mean cell area of adipocytes (*n* = 5 per group). Data are presented as the mean ± SEM and analyzed using *t* test. *, *P* < 0.05; **, *P* < 0.01. *n* = 8 to 9 for both groups for all analyses, except *n* = 5 for panel h.

Considering that the ketogenic diets used in the above two trials were nearly devoid of carbohydrates, the ketogenic diets used in human studies permit low carbohydrate consumption (approximately 5% to 10% of total caloric intake or below 50 g per day) (19). Thus, we customized another ketogenic diet called KD(72%) with 72% fat, 20% protein, and 8% carbohydrate. Notably, to eliminate the influence of TFAs in diet, KD(72%) used the same sources of nutrients as KDH. We observed increased β-hydroxybutyrate concentrations in the KD(72%) mice [NC, 0.44; KD(72%), 0.8 mmol/liter; *P* < 0.001]. Moreover, KD(72%) damaged metabolic health, which was mainly reflected in two aspects. First, although the fasting blood glucose level and glucose clearance during OGTT in the KD(72%) mice were similar to those in the NC group (Fig. 6a and b), the secretion of insulin was significantly higher in the KD(72%) mice (Fig. 6c and d), and the KD(72%) mice also had a higher HOMA-IR index and insulin resistance index (Fig. 6e and f). This suggests that insulin sensitivity was reduced in the KD(72%) mice. Second, the KD(72%) mice showed significantly higher fat mass and larger adipocyte size than the NC mice (Fig. 6g and h). To summarize, these results showed that KD(72%) induced insulin resistance and fat accumulation in mice.

**FIG 7:**
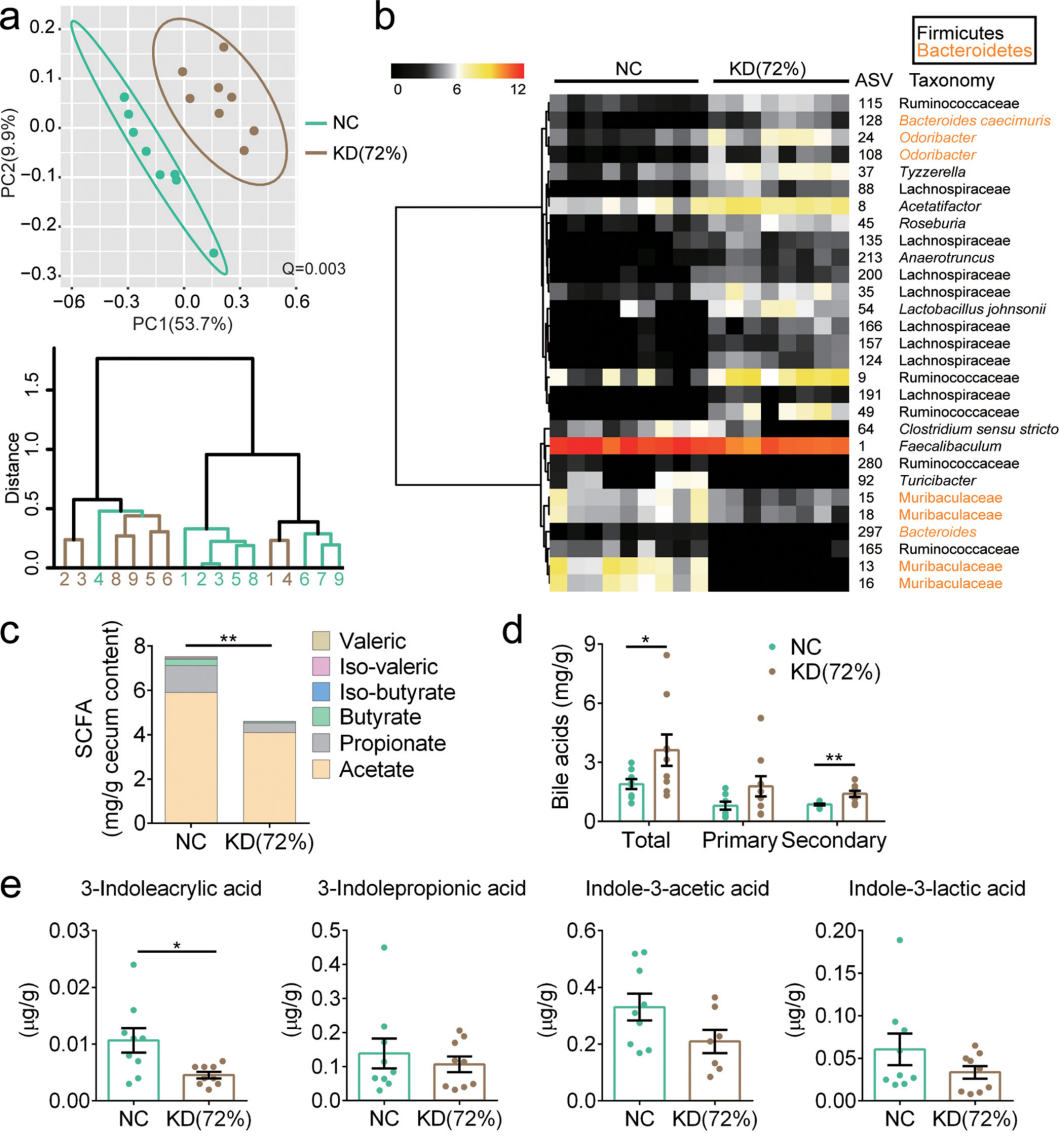
KD(72%) altered the gut microbiota and metabolites in mice. (a) Top, PCoA plot of gut microbiota based on the Bray-Curtis distance. Bottom, clustering of the gut microbiota between different groups calculated with PERMANOVA (9,999 permutations) using Bray-Curtis distances. (b) The heatmap represents the normalized and log_2_-transformed relative abundance of the 29 ASVs significantly different between two groups. These ASVs were clustered by the ward.D method. (c) The concentrations of SCFAs in cecum content of mice (*n* = 5 to 6 per group). (d) The concentrations of BAs in cecum content of mice. (e) The concentrations of tryptophan metabolites in fecal samples of mice. Data are presented as the mean ± SEM and analyzed using *t* test. *, *P* < 0.05; **, *P* < 0.01. *n* = 8 to 9 for both groups for all analyses, except *n* = 5 to 6 for panel c.

By 16S rRNA gene sequencing, we found that the KD(72%) mice displayed significantly altered gut microbiota compared to the NC group (*P* < 0.01 with PERMANOVA, 9,999 permutations) (Fig. 7a). Although selected specific ASVs were not exactly the same (Fig. S7), KD(72%) still enhanced the abundance of *Roseburia*, *Ruminococcaceae*, and *Lachnospiraceae* and reduced the abundance of *Turicibacter* similarly to the KDs in the above-mentioned trials (Fig. 7b). Furthermore, significantly decreased SCFAs and increased BAs levels were also observed in the KD(72%) mice (Fig. 7c and d). Although the concentration of 3-indoleacrylic acid was significantly decreased in the KD(72%) mice, there was no significant difference in the concentrations of other tryptophan metabolites compared to those in the NC group (Fig. 7e). Taken together, these results suggested that KD(72%) impaired metabolic health, gut microbiota, and metabolites in mice.

10.1128/mBio.03601-20.7 FIG S7 Thirty-eight ASVs differentiating gut microbiota structure between NC and KD(72%) mice. (a) The sPLS-DA plot of gut microbiota. (b) The classification error rate of one component across the leave-one-out cross-validation for maximum distance. (c) The heatmap of selected 38 ASVs between NC and KD(72%) mice. Download FIG S7, PDF file, 0.3 MB. Copyright © 2021 Li et al. 2021 Li et al. https://creativecommons.org/licenses/by/4.0/ This content is distributed under the terms of the Creative Commons Attribution 4.0 International license.

## DISCUSSION

In the current study, we showed that the impaired glucose and lipid metabolism in mice induced by KDs may be due to the sources and proportions of fat in these diets, which are associated with their distinct effects on gut microbiota and metabolites.

The KDs, regardless of the proportions and sources of fat, impaired glucose and lipid metabolism in mice in our study, which is in agreement with previous reports that KDs caused glucose intolerance, insulin resistance, hepatic steatosis, and fat accumulation (11–17). Several studies had reported the possible mechanisms of glucose intolerance and insulin resistance induced by KDs, such as the reduction in γδ T cells that can restrain inflammation (18), or the decreased cell surface expression of GLUT4 in skeletal muscle, which reduces glucose uptake in the periphery (11). Regarding the hepatic lipid accumulation induced by KD, Schugar et al. reported that the synergistic effects of protein restriction and choline deficiency may be potential contributors (23). Notably, an impaired colonic mucus barrier has been suggested as one of the causes of metabolic damage (24), and the expression of genes related to the intestinal barrier (*Occludin*, *Muc2*) was significantly reduced by 5 weeks of KD consumption in our work. Although improved serum glucose and reduced weight were reported in short-term (1-week) KD studies (6, 18, 20), our work observed that extending feeding time with KD impaired host metabolism, especially in glucose tolerance and hepatic lipid accumulation. The glucose and lipid metabolism disorders are a gradual accumulation process, and our work emphasizes that there is a risk of metabolic disorders on a long-term KD. Future work is still needed to identify the overall mechanism by which KDs impair glucose and lipid metabolism.

The destruction of glucose and lipid metabolism induced by KDs may be partly related to the changes in the gut microbiota and metabolites. In the current work, both two types of KD induced the changes of gut microbiota and metabolites. For example, we found that the increase in the abundance of many ASVs belonging to *Ruminococcaceae* and *Lachnospiraceae* was correlated with impaired lipid metabolism induced by two formulas of KD. Previously, a strain belonging to *Lachnospiraceae* was reported to induce the development of obesity and diabetes in germfree ob/ob mice (25). However, we also observed that the abundance of few ASVs belonging to *Lachnospiraceae* decreased in KD-fed mice, indicating that the response of gut microbiota to KD is species and strain specific, and this may explain our inconsistency with other studies (21). Furthermore, 3 ASVs belonging to *Bacteroides* were enriched only in our KDR mice, which could convert primary BAs into secondary BAs through the production of bile salt hydrolase (BSH) (26). Recent studies reported that transplantation of Bacteroides vulgatus resulted in insulin resistance in recipient mice (27). In addition, a high secondary bile acid DCA level was observed in our KDR mice. DCA could bind to farnesoid X receptor (FXR), and the activation of FXR could induce metabolic disorders (28). Thus, KDR induces severe glucose intolerance and insulin resistance in mice, which may be due to increased abundance of *Bacteroides* and high DCA level. Notably, we found that the abundance of Lactobacillus murinus was decreased in the mice with KD. In our previous studies, we found abundant Lactobacillus murinus in the mice with calorie restriction (CR) (29, 30), which was significantly correlated with the improvement of midlife metabolic phenotypes. Moreover, we isolated a strain, *L. murinus* CR147, from the feces of CR mice and showed that this strain reduced intestinal permeability and systemic inflammatory markers in old microbiota-colonized mice (30). The beneficial effects of Lactobacillus murinus on glucose and lipid metabolism may be related to the ability to produce SCFAs and convert tryptophan into indole derivatives (31). SCFAs can protect the intestinal barrier by increasing the secretion of glucagon-like peptide-2 (GLP-2) (32) and exert anti-inflammatory effects by binding the G-protein-coupled receptor 41/43 (GPR41/43) (33). IAA was reported to play a role as aryl hydrocarbon receptor (AhR) in regulation of intestinal health (34). IPA can decrease intestinal permeability mediated by the pregnane X receptor (PXR) (35). The impaired intestinal barrier and an inflammatory condition have been regarded as a crucial pathological process underlying metabolic disorders (22, 36). In our work, decreased levels of SCFAs and tryptophan metabolites were observed in the mice with KD. Therefore, the decreased abundance of Lactobacillus murinus induced by two formulas of KD may contribute to the impaired glucose and lipid metabolism. Our studies pointed out that candidate bacteria and their potential metabolites correlated with KD-induced metabolic disorders. In future studies, we should use these specific bacteria and their metabolites responding to KD to investigate its causality in metabolic disorders and involved mechanism.

More importantly, we showed that both the sources and proportions of fat in the KDs modified glucose and lipid metabolism in mice, suggesting that the controversial results from previous reports may be due to neglect of the composition of the KD. We found that KDR but not KDH induced glucose intolerance and insulin resistance. A recent study using the same KD as our KDR demonstrated that long-term KD feeding depleted adipose-resident γδ T cells, leading to obesity and the loss of glycemic regulation in mice (18). Previous studies using a diet consistent with our KDH showed that KD reduced serum glucose in animal models (20, 37). In our study, we analyzed the composition of these two formulas of KD and found that their sources of fat are very different. Therefore, future work in animal and clinical studies related to diet and nutritional intervention should consider not only the proportion of nutrients but also the source.

For the diets used in animal trials, the source of them may have an impact on the experimental results, especially in metabolic phenotypes. For example, KDR-induced severe glucose intolerance in mice may be due to high TFAs levels in fat. In epidemiological investigations, TFA intake was associated with an increased risk of cardiovascular disease and incidence of obesity and type 2 diabetes mellitus (38, 39). And in a mouse model, high TFA intake resulted in decreased sensitivity of insulin receptor substrate 1 (IRS1) and downregulation of adipose triglyceride lipase (ATGL) expression in the liver (40). In an *in vitro* model, trans-9-octadecenoic acid may reduce the glucose uptake by cells through suppressing insulin-induced elevations of phosphorylated Akt levels and the fusion of GLUT4 storage vesicles (41). Our KDR (Research Diets D12369B) is a KD commonly used in animal trials, and the experimental results of the previous studies using this diet cannot be attributed to high fat content completely but may also be related to TFAs in fat.

Overall, our study demonstrated that the metabolic disorders induced by KDs are closely related to the source and proportion of fat in the diet, which may be associated with the changes of the gut microbiota and metabolites. Although KDs have beneficial effects on epilepsy and neurodegenerative diseases (1), according to our findings, KDs truly impair glucose and lipid metabolism. Thus, when using these diets for weight loss and the treatment of other conditions, the risk of glucose and lipid metabolism disorders should be kept in mind, and the sources and proportions of fat in these diets need to be further evaluated.

## MATERIALS AND METHODS

### Ethical approval.

All animal experimental procedures were approved by the Institutional Animal Care and Use Committee (IACUC) of Shanghai Jiao Tong University (no. 2018043 and 2018106).

### Animal trial.

The mice were purchased from SLAC, Inc. (Shanghai, China), and the experiments were performed at the animal center of Shanghai Jiao Tong University. All mice were kept under a standard 12-h light/dark cycle (lights on at 7:00 a.m.) and a temperature of 22°C ± 3°C.

### (i) Trial 1.

After 2 weeks of accommodation, 8-week-old specific-pathogen-free (SPF) C57BL/6J male mice were randomly assigned into four groups (9 mice per group) and fed two kinds of ketogenic diets or their control normal chow for 5 weeks: (i) fed ketogenic diet type 1 (KDR; Research Diets D12369B, 0.1% carbohydrate, 10.4% protein, 89.5% fat, 6.76 kcal/g), (ii) fed control normal chow of KDR (NCR; Research Diets D12450J; 70% carbohydrate, 20% protein, 10% fat, 3.85 kcal/g), (iii) fed ketogenic diet type 2 (KDH; 1% carbohydrate, 7.7% protein, 91.3% fat, 6.77 kcal/g, produced by FBSH Biotechnology Ltd., Shanghai, China), or (iv) fed control normal chow of KDH (NCH; 64.5% carbohydrate, 20% protein, 15.5% fat, 4 kcal/g, produced by FBSH Biotechnology Ltd., Shanghai, China). The formulas of these diets are shown in Table S1. The mice were allowed *ad libitum* access to water and food. Both the KDR and KDH mice were fed freshly prepared ketogenic diets every day because of the perishability of ketogenic diets. The body weight of the mice and food intake per cage (3 mice per cage) were measured every 3 days.

### (ii) Trial 2.

After 2 weeks of acclimation, 8-week-old SPF C57BL/6J males were randomly assigned into three groups (9 mice per group) and fed ketogenic diets with two different proportions of fat or normal chow for 5 weeks: (i) fed normal chow (NCH), (ii) fed KDH, or (iii) fed a ketogenic diet containing 89.5% fat [KD(89.5%); 0.1% carbohydrate, 10.4% protein, 89.5% fat, 6.57 kcal/g; produced by FBSH Biotechnology Ltd., Shanghai, China]. The formulas of these diets are shown in Table S1. The mice were allowed *ad libitum* access to water and food. Both the KDH and KD(89.5%) mice were fed freshly prepared ketogenic diets every day. The body weight of the mice and food intake per cage (3 mice per cage) were measured every 3 days.

### (iii) Trial 3.

After 2 weeks of accommodation, 8-week-old SPF C57BL/6J males were randomly assigned into two groups (9 mice per group) and fed a ketogenic diet or normal chow for 5 weeks: (i) fed normal chow (NC; same as NCH in the above-mentioned trial) or (ii) fed a ketogenic diet containing 72% fat [KD(72%); 8% carbohydrate, 20% protein, 72% fat, 5.65 kcal/g; produced by FBSH Biotechnology Ltd., Shanghai, China]. The formulas of these diets are shown in Table S1. The mice were allowed *ad libitum* access to water and food. The KD(72%) mice were fed a freshly prepared ketogenic diet every day. The body weight of the mice and food intake per cage (3 mice per cage) were measured every 3 days.

### Histopathology of eAT, liver, pancreas, and ileum.

For epididymal adipose tissue (eAT) and liver, fresh tissue was fixed with 4% paraformaldehyde, embedded in paraffin, sectioned (4-μm thickness), and stained with hematoxylin and eosin (H&E) (Wuhan Servicebio Technology Ltd., Wuhan, China). Adipocyte size of eAT sections was assessed using Image-Pro Plus v6.0 (Media Cybernetics Inc., Silver Spring, MD, USA), and the mean cell area of adipocytes was determined in at least three discontinuous scans (>300 total adipocytes) in five random mice from each group. The histologic scores (NAFLD activity score) of liver sections were assessed as described previously (42). For the pancreas, fresh tissue was fixed with 4% paraformaldehyde, embedded in paraffin, sectioned (4-μm thickness), and stained for insulin (green) after standard indirect immunofluorescence staining. The islet areas were determined in at least three discontinuous scans under ×400 magnification and examined as the total islet areas divided by the total number of islets. For the ileum, immunohistochemistry staining for ZO-1 was performed as previously described (29).

### Blood ketone measurement.

After 6 h of fasting, blood ketone (β-hydroxybutyrate) levels were measured in blood samples collected from the tip of the tail vein with a blood ketone meter (FreeStyle Optium Neo; Abbott, USA).

### Oral glucose tolerance test.

After 6 h of fasting, mice were administered glucose (2 g/kg body weight) by oral gavage, and blood samples were collected from the tip of the tail vein at 0, 15, 30, 60, 90, and 120 min after glucose administration for assessment of glucose concentrations using a blood glucose meter (Accu-Chek Performa; Roche, USA). Blood samples were collected from the tail vein into tubes at 0, 15, and 60 min after glucose administration to determine insulin concentrations. The degree of insulin resistance was estimated by calculating the HOMA-IR (homeostatic model assessment for insulin resistance, glucose × insulin/22.5 × 21.2 from fasting samples) index and insulin resistance index (the product of the 0- to 60-min area under the curve [AUC] of blood glucose and that of serum insulin in OGTT).

### Serum LBP and insulin measurement.

After blood was placed at room temperature for 40 min, the samples were centrifuged at 3,500 rpm for 15 min at 4°C, and the supernatant was collected and then stored at −80°C until further analyses. According to the manufacturer’s instructions, enzyme-linked immunosorbent assays (ELISAs) were used to quantify lipopolysaccharide (LPS)-binding protein (LBP) (PCDBM0177; P&C Biotechnology Ltd., China) and insulin (90080; Chrystal Chem, USA).

### Hepatic lipid measurement.

Semithawed liver tissue samples were cut to approximately 0.1 g and homogenized in a corresponding volume (1/9, wt/vol) of homogenizing buffer (pH 7.4; 0.01 mol/liter Tris-HCl, 0.1 mmol/liter EDTA-Na_2_, 0.8% NaCl) in screw tubes. Next, the tubes were shaken for 5 min at 25 Hz/s with a TissueLyser (Qiagen, Germany) and then put into an ice water mixture for 5 min, and the above steps were repeated again. Afterward, the supernatant was collected after being centrifuged at 2,500 rpm for 10 min at 4°C and used to measure concentrations of hepatic total cholesterol (A111-1-1; Nanjing Jiancheng Bioengineering Institute, Nanjing, China) and triglyceride (A110-1-1; Nanjing Jiancheng Bioengineering Institute, Nanjing, China) using the assay kits. The hepatic lipid results were corrected by calculating the total protein concentration (20202ES76; Yeasen Biotechnology Ltd., China).

### Colon RNA isolation and reverse transcription qPCR (RT-qPCR).

According to the manufacturer’s protocol, total RNA was extracted from the colon using an RNeasy minikit (74804; Qiagen, Germany) and reverse transcribed into cDNA using a SuperScript kit (18080-051; Invitrogen, USA). Quantitative real-time PCR (qPCR) was performed in an 20-μl reaction system containing template, forward and reverse primers, and SYBR green I PCR Supermix (Bio-Rad) on a LightCycler 96 system (Roche Applied Sciences) and used to assess the expression of *Zo-1*, *Occludin*, *Muc1*, *Muc2*, and *Muc3*. The PCR conditions were 95°C for 3 min, followed by 40 cycles of 95°C for 20 s, 56°C for 30 s, and 72°C for 30 s, and plate reads for 5 s. Gene expression levels were determined using the cycle threshold (2^−ΔΔ^*^CT^*) method, with *Beta-actin* serving as the reference gene. Forward (F) and reverse (R) primer sequences (43) are as follows: *Zo-1*, F-ACCCGAAACTGATGCTGTGGATAG and R-AAATGGCCGGGCAGAACTTGTGTA; *Occludin*, F-ATGTCCGGCCGATGCTCTC and R-TTTGGCTGCTCTTGGGTCTGTAT; *Muc1*, F-TACCCTACCTACCACACTCACG and R-CTGCTACTGCCATTACCTGC; *Muc2*, F-CACCAACACGTCAAAAATCG and R-GGTCTCTCGATCACCACCAT; *Muc3*, F-CTTCCAGCCTTCCCTAAACC and R-TCCACAGATCCATGCAAAAC; *Beta-actin*, F-GGCTGTATTCCCCTCCATCG and R-CCAGTTGGTAACAATGCCATGT.

### Statistical analysis for animal trials.

Statistical significance for physiological and biochemical data of mice in different groups was analyzed with one-way analysis of variance (ANOVA) followed by a Tukey *post hoc* test using the “stats” and “multcomp” packages in R (v3.6.1) (www.r-project.org). Differences were considered statistically significant when *P* was <0.05.

### qPCR of total fecal bacteria.

A plasmid of the 16S full-length positive Akkermansia muciniphila strain (*n* = 10^9^ copies/μl) was diluted according to different gradients successively to 10^8^, 10^7^, 10^6^, 10^5^, 10^4^, 10^3^, and 10^2^ copies/μl. qPCR was performed in a 20-μl reaction system containing template (20 ng), primer Uni331F (5′-TCCTACGGGAGGCAGCAGT-3′), primer Uni797R (5′-GGACTACCAGGGTATCTAATCCTGTT-3′) (44), and supermix (Bio-Rad) on a LightCycler 96 system, with 2 replicates for standard and sample DNA. The PCR conditions were 95°C for 3 min, followed by 40 cycles of 95°C for 15 s, 60°C for 60 s, and 80°C for 5 s, and plate reads for 5 s. A standard curve was determined though a linear fit of the copy number and *C_T_* value of the plasmid in different gradients. The copy number of sample DNA was calculated through a standard curve using Opticon Monitor 3.1. Data were analyzed with one-way ANOVA followed by a Tukey *post hoc* test.

### 16S rRNA gene V3-V4 region sequencing and data analysis.

During the animal trial, fresh feces were collected weekly and stored at −80°C until analysis. DNA was extracted from fecal samples collected at the 5th week using a previously described method (45). A sequencing library of the V3-V4 regions of the 16S rRNA gene was constructed according to the manufacturer’s instructions (part no. 15044223 Rev. B; Illumina Inc., CA, USA) with some modifications as previously published (46) and sequenced on the MiSeq system (Illumina, Inc., CA, USA) with the MiSeq reagent kit v3 (2 × 300 cycles, no. MS-102-3033).

The raw paired-end reads were submitted to Quantitative Insights Into Microbial Ecology2 (QIIME2, v 2018.11) (47) for analysis. Sequence quality and primer removal were processed using the “Demux” and “cutadapt” plugins, respectively, and DADA2 (48) was used to obtain a quality-filtered, denoised, chimera-free, and merged amplicon sequence variants (ASVs) (49). Representative sequences for each ASVs were built into a phylogenetic tree by FastTree and assigned taxonomic classifications using the SILVA132 16S rRNA database (50). Alpha diversity metrics (observed ASVs and Shannon index), beta diversity metrics, and principal-coordinate analysis (PCoA) were calculated using the “core-metrics-phylogenetic” plugin after sequencing depth was downsized to 20,000 (trial 1) and 13,000 (Trials 2 and 3) sequences per sample. The statistical significance of gut microbiota among different groups was assessed by permutational multivariate analysis of variance (PERMANOVA; 9,999 permutations). PERMANOVA clustering analysis was performed with Bray-Curtis distances. Differences were considered significant when *Q* was <0.05.

Sparse partial least-squares discriminant analysis (sPLS-DA) (51) was performed using the “mixOmics” (v6.8.2) package (52) in R to identify the discriminative ASVs between different groups (NCR and KDR, NCH and KDH, and KDR and KDH). The optimal classification performance of the sPLS-DA model was assessed with the perf function using leave-one-out cross-validation with the smallest error rate. Variations in the relative abundance of selected ASVs in different groups were analyzed by the Mann-Whitney U test in MATLAB (R2017b). Differences were considered significant when *P* was <0.05.

The visual presentation of the heatmap showing the relative abundance of selected ASVs in different groups was performed using the “pheatmap” package in R. Spearman correlations between ASV abundance and host parameters related to glucose and lipid metabolism were calculated using MATLAB. The *P* values were adjusted by false discovery rate (FDR) estimation using the Benjamini-Hochberg method (53). The visual presentation of correlations was performed using the “ggcorrplot” package in R. Correlations were considered significant when FDR was <0.05.

### Untargeted metabolomics study and data analysis.

Cecum content samples (60 mg) were homogenized in 200 μl distilled water and vortexed. Next, 800 μl of methanol-acetonitrile solution (1:1, vol/vol) mixture was added to precipitate protein, pulverized by ultrasonic wave at low temperature for 30 min, and then kept at −20°C for 1 h. Next, samples were centrifuged at 14,000 relative centrifugal force (rcf) for 20 min at 4°C and stored at −80°C after removing the supernatant and vacuum drying. Quality control (QC) samples were prepared using the same methods. Ultra-high-performance liquid chromatography–quadrupole time-of-flight mass spectrometry (UHPLC-Q-TOF-MS) analysis of cecal contents in mice was performed by Shanghai Applied Protein Technology Ltd. (Shanghai, China) on the 1290 Infinity ultrahigh-performance liquid chromatography system (Agilent, Germany) coupled with a Triple TOF 5600 system (AB Sciex, USA). The raw data were converted into mzXML format using Proteo Wizard software, and peak alignment, retention time correction, and peak area extraction were performed using the XCMS program. Details of the data processing and analysis were performed as described previously (54).

Variable importance in projection (VIP) scores obtained from the orthogonal projection to latent structure-discriminant analysis (OPLS-DA) model were used to assess the contribution of variables. Metabolites that had a VIP of >1 between different groups (NCR and KDR, NCH and KDH, and KDR and KDH) were defined as discriminating metabolites, and then these discriminating metabolite levels were compared between different groups using Student’s *t* test statistical analysis. Only discriminating metabolites that had *P* of <0.05 were defined as significant discriminative metabolites and identified by searching an in-house standard MS/MS library (using exact mass data [mass error ≤ 25 ppm] or MS/MS spectra matching).

The visual presentation of the heatmap showing the standardized concentration of significant discriminative metabolites in different groups was performed using the “pheatmap” package in R. Spearman correlations between metabolite concentration and host parameters related to glucose and lipid metabolism were calculated using MATLAB. The *P* values were adjusted by FDR estimation. The visual presentation of correlations was performed using the “ggcorrplot” package in R. Correlations were considered significant with an FDR of <0.05.

The “effect size” analysis strategy (55) was used to determine the biomarker metabolites affecting the gut microbiome. The adonis function of the “vegan” package in R was used to estimate the “one-to-all” effect size (*R*^2^) between each single variable of metabolites to the whole gut microbiome. Only variables with significant (*P* < 0.05, 999 permutations) effects and *R*^2^ > 0.09 on the gut microbiome were considered later.

### Targeted fatty acid quantitation.

Gas chromatography-mass spectrometry (GC-MS) analysis of fatty acids in the three ketogenic diets [KDR, KDH, and KD(89.5%); 3 replicates for each diet] was performed by BioNovoGene Technology Ltd. (Suzhou, China) on a GC-MS-QP2010 Ultra system (Shimadzu, Japan). Data were analyzed with one-way ANOVA followed by a Tukey *post hoc* test. Samples (200 to 400 mg) were homogenized in a 1-ml *N*-hexane–isopropanol solution (3:2, vol/vol) mixture with 3-mm-diameter zirconia/silica steel beads, pulverized by ultrasonication for 30 min, and then centrifuged at 12,000 rpm for 5 min. Then, 500 μl of supernatant was added to 1 ml *N*-hexane. After repeating centrifugation for 5 min, 900 μl of supernatant was added to 400 μl methanol. After pulverizing in an ultrasonic wave for 5 min, 40 μl diazomethane was added and kept at room temperature for 15 min, and then centrifugation was repeated. Two hundred microliters of *N*-hexane was added after collecting the supernatant (200 μl) and vacuum drying, and the samples were centrifuged. Finally, the supernatant was collected and transferred into a GC vial for GC-MS analysis.

### Targeted short-chain fatty acid (SCFA) quantitation.

GC-MS analysis of SCFAs in the cecal contents of mice was performed on an Agilent 6890 (Agilent Technologies, CA, USA) with flame ionization, thermal conductivity detectors, and capillary columns. Data were analyzed with one-way ANOVA followed by a Tukey *post hoc* test. Then, 50 mg cecal contents was homogenized with 250 μl phosphate buffer and centrifuged at 16,000 × *g* for 15 min at 4°C. The supernatants were filtered through 0.22-μm filters. Eighty microliters of supernatant was acidified by adding 40 μl of 50% (vol/vol) sulfuric acid. After vortexing and standing for 2 min, the organic acids were extracted by adding 160 μl of diethyl ether. Finally, the supernatant was collected for GC-MS analysis.

### Targeted bile acid quantitation.

Ultra-high-performance liquid chromatography–mass spectrometry (UPLC-MS) analysis of bile acids in the cecal contents of mice was performed on a Waters Acquity UPLC using an ethylene-bridged hybrid (BEH) C_18_ column (1.7 μm, 2.1 × 100 mm) coupled with a Triple TOF 4000 system equipped with an electrospray ionization (ESI) source operating in negative mode (AB Sciex, USA). Data were analyzed with one-way ANOVA followed by a Tukey *post hoc* test. Ten milligrams of cecal contents from mice was preweighed, mixed with 1 ml precooled methanol (−20°C) and 100 mg glass beads, and shaken for 1 min at 25 Hz/s, and this process was repeated at least two times. Next, samples were pulverized by ultrasonic wave for 30 min and then centrifuged at 12,000 rpm for 10 min at 4°C. Methanol (580 μl) was added after collecting the supernatant (20 μl) and vortexed for 30 s. Finally, the supernatant was collected for LC-MS analysis.

### Targeted tryptophan metabolite quantitation.

UPLC-MS analysis of tryptophan metabolites in the feces of mice was performed on a Waters Acquity UPLC using an HSS T3 column (1.8 μm, 2.1 × 100 mm) coupled with a Q Exactive mass spectrometer equipped with an ESI source operating in negative mode (Thermo, USA). Data were analyzed with one-way ANOVA followed by a Tukey *post hoc* test. Thirty milligrams of feces from mice was preweighed and mixed with 500 μl of 50% acetonitrile, followed by thorough homogenization. Next, samples were kept on ice for 30 min and centrifuged at 12,000 rpm for 10 min at 4°C. The supernatant was collected, and the above steps were repeated three times. Two hundred microliters of 50% acetonitrile was added after collecting the supernatant and vacuum drying and centrifuged at 12,000 rpm for 10 min. Finally, the supernatant was collected for LC-MS analysis.

### Data availability.

The raw Illumina sequence data generated during the current study have been deposited to the NCBI under BioProject accession no. PRJNA674951.
