# Supplementary figures and images for: Ketogenic Diets Induced Glucose Intolerance and Lipid Accumulation in Mice with Alterations in Gut Microbiota and Metabolites
Source: mBio. 2021 Mar 30;12(2):e03601-20. doi: 10.1128/mBio.03601-20 (PMC8092315; doi:10.1128/mBio.03601-20)

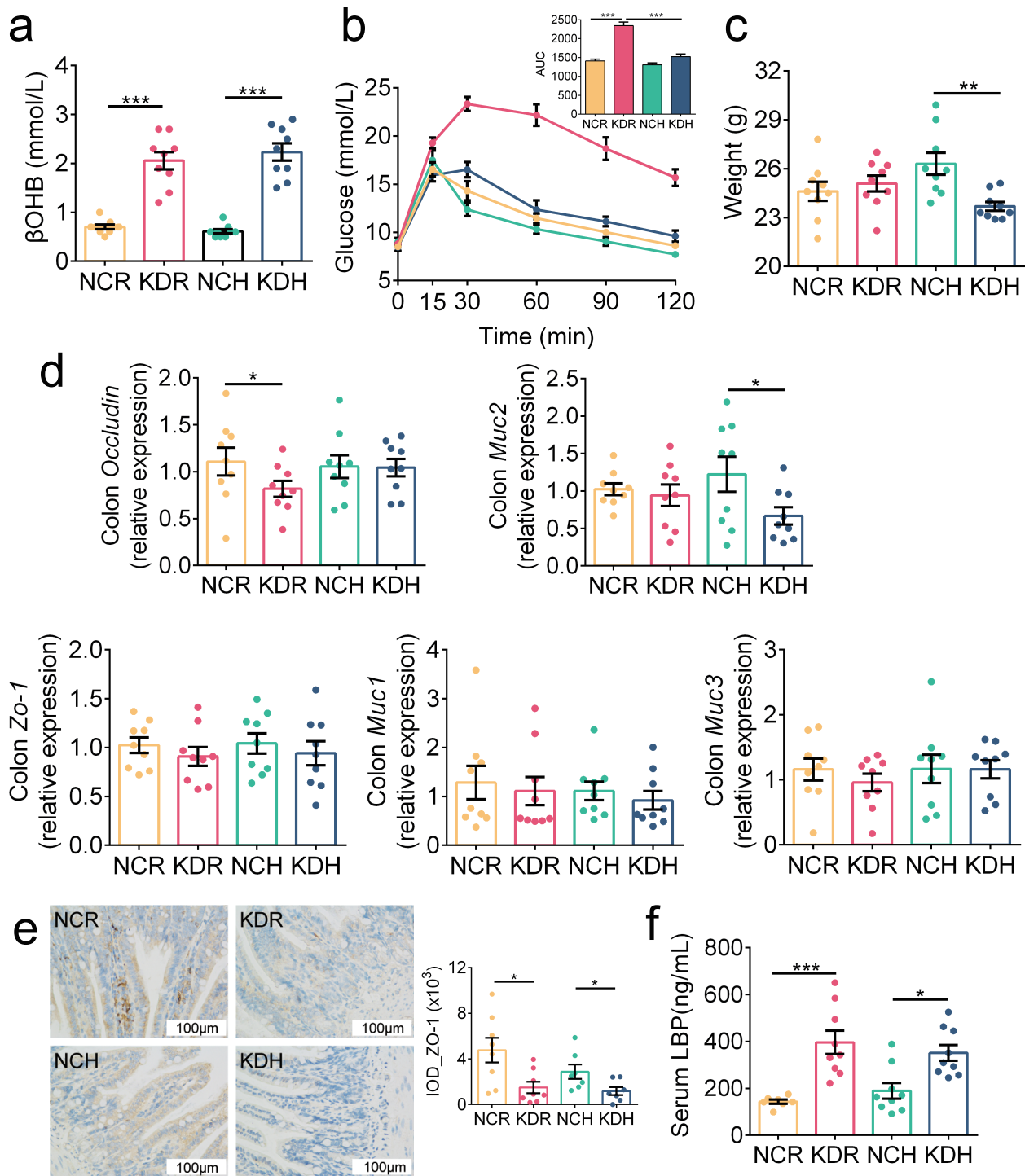

Supplement: FIG S1 [file mBio.03601-20-sf001.pdf]

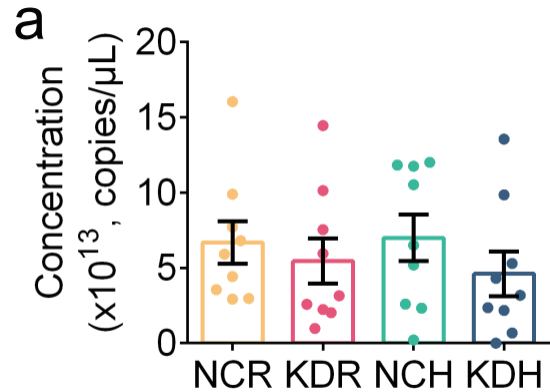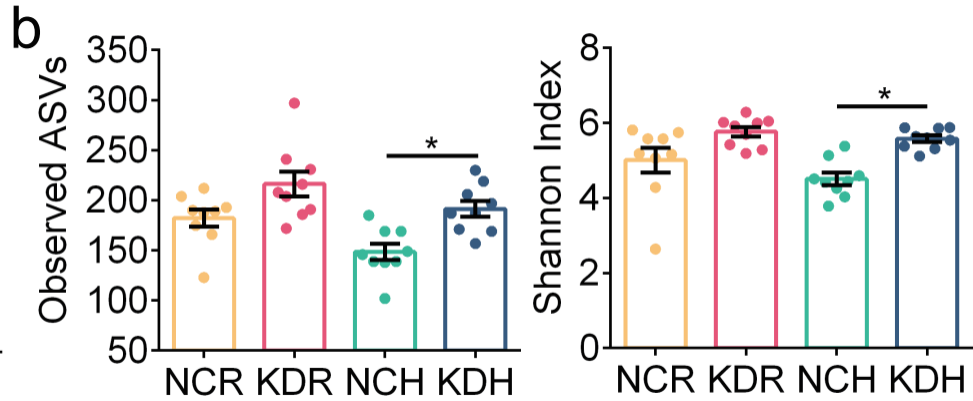

Supplement: FIG S2 [file mBio.03601-20-sf002.pdf]

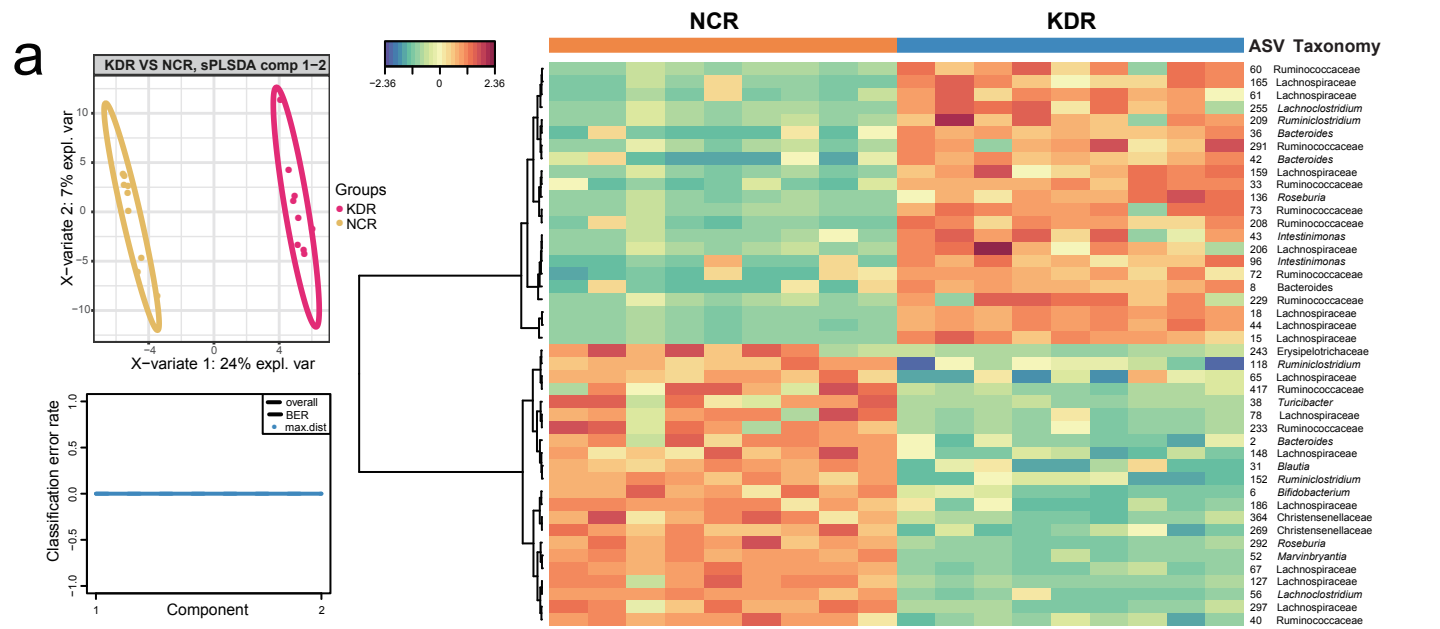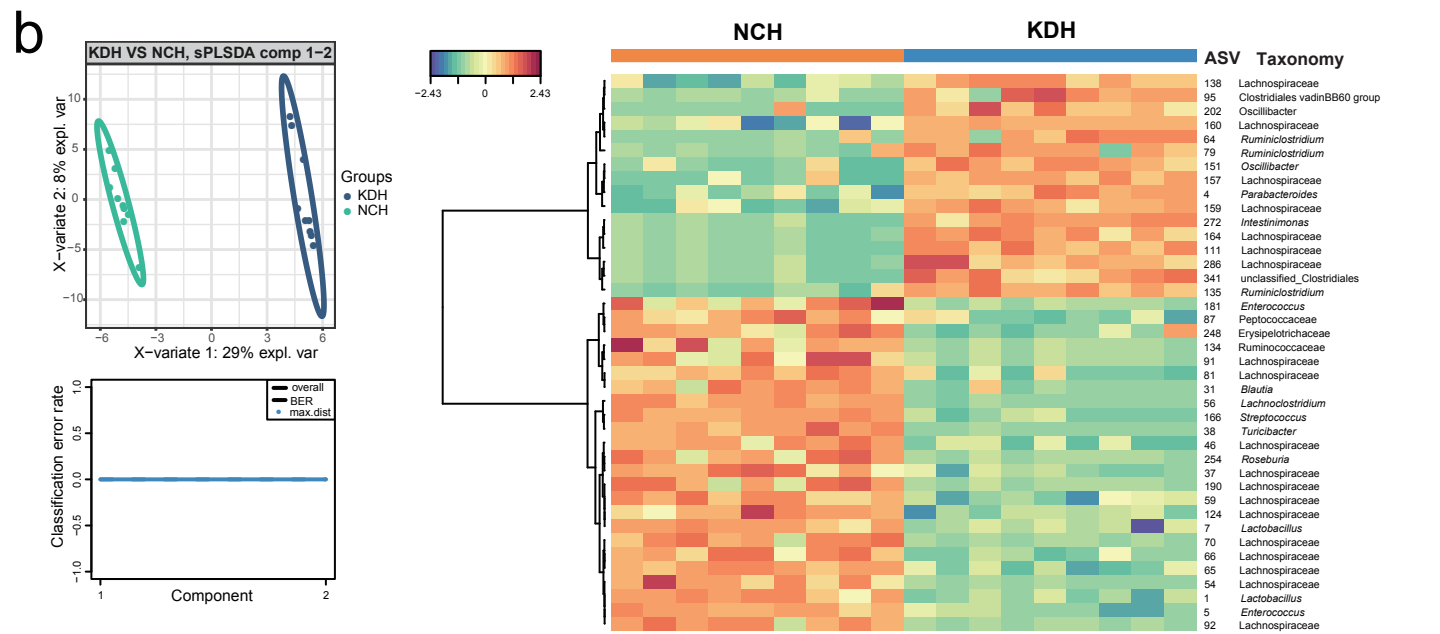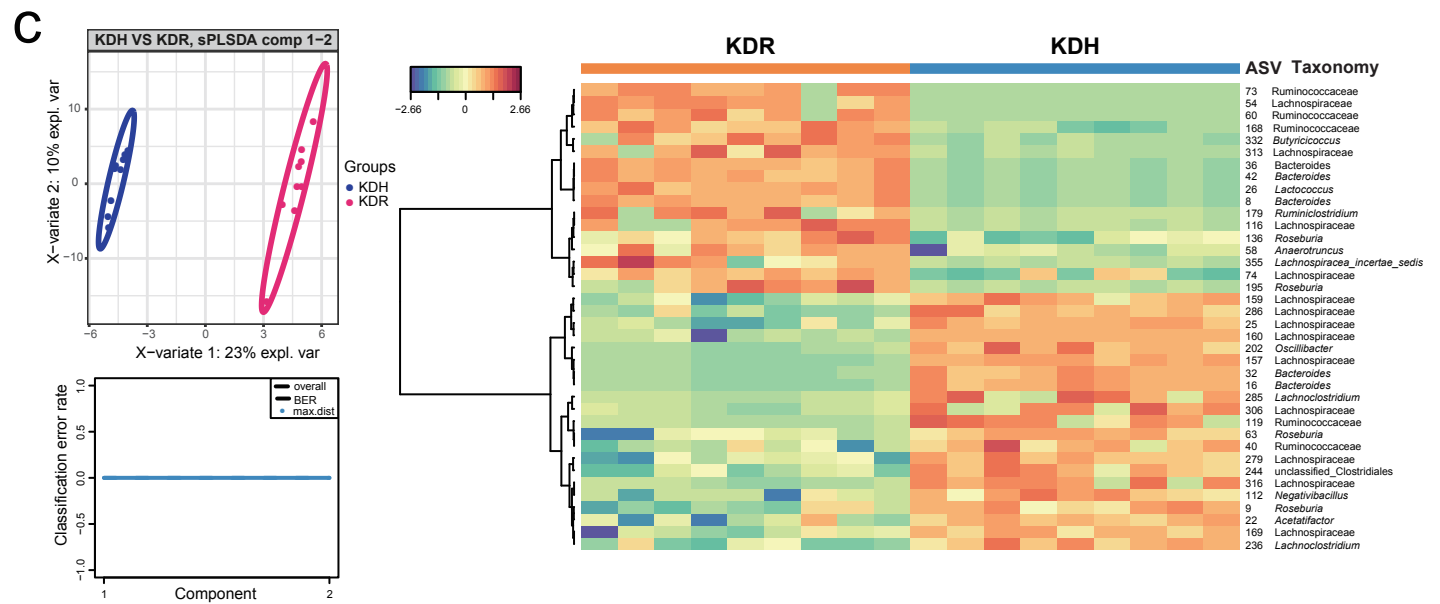

Supplement: FIG S3 [file mBio.03601-20-sf003.pdf]

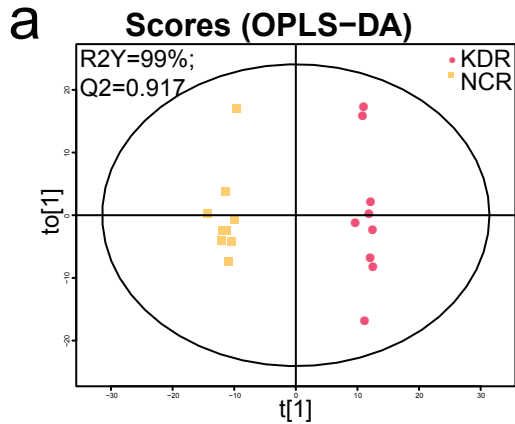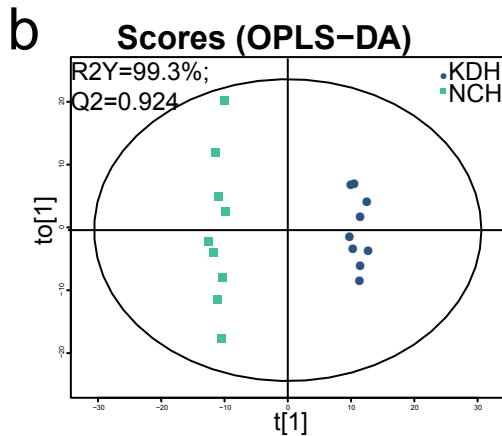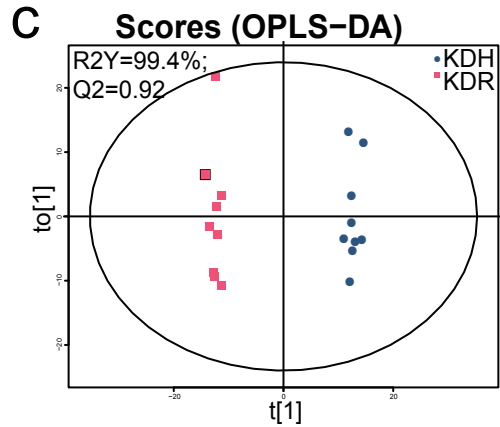

Supplement: FIG S4 [file mBio.03601-20-sf004.pdf]

trans-6-Octadecenoic acid

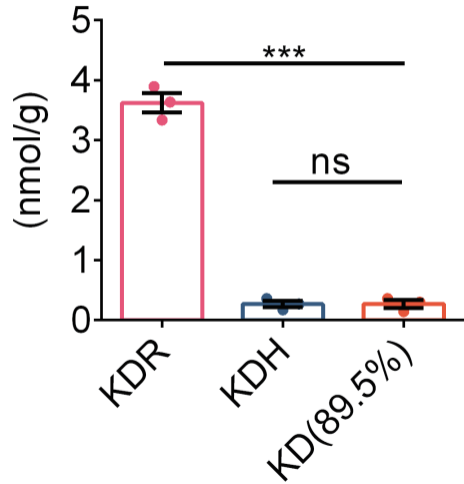

trans-9-Octadecenoic acid

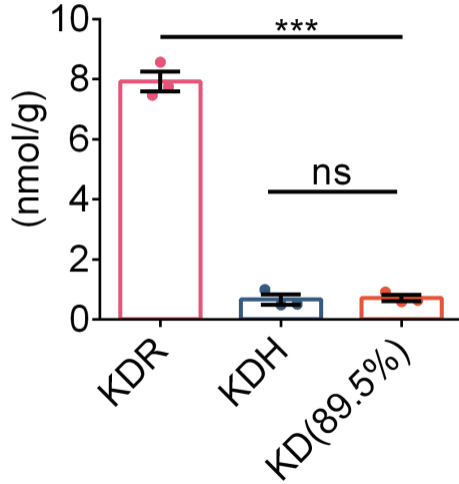

trans-11-Octadecenoic acid

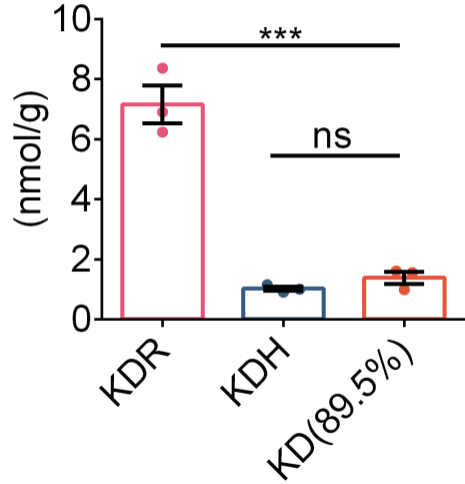

Supplement: FIG S5 [file mBio.03601-20-sf005.pdf]

a

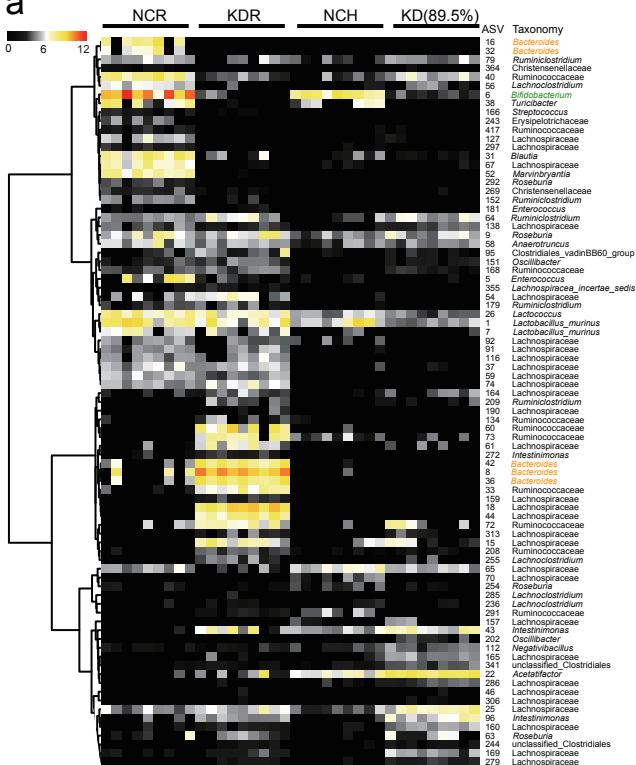

b

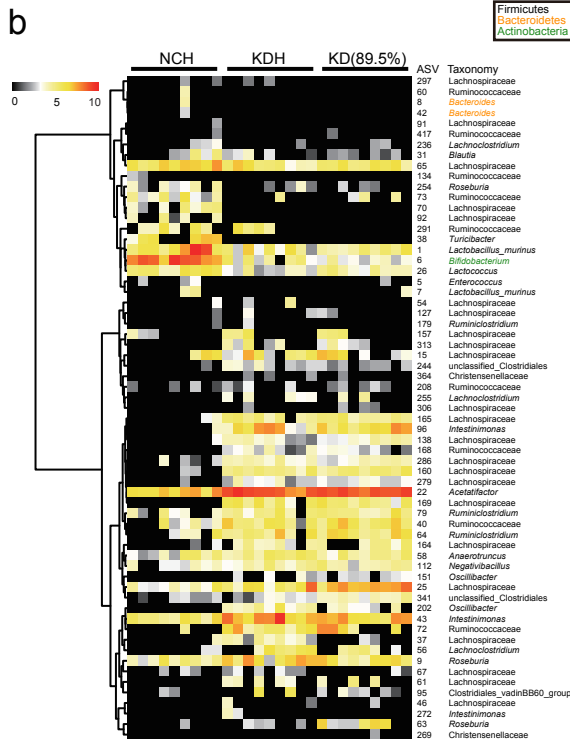

Supplement: FIG S6 [file mBio.03601-20-sf006.pdf]

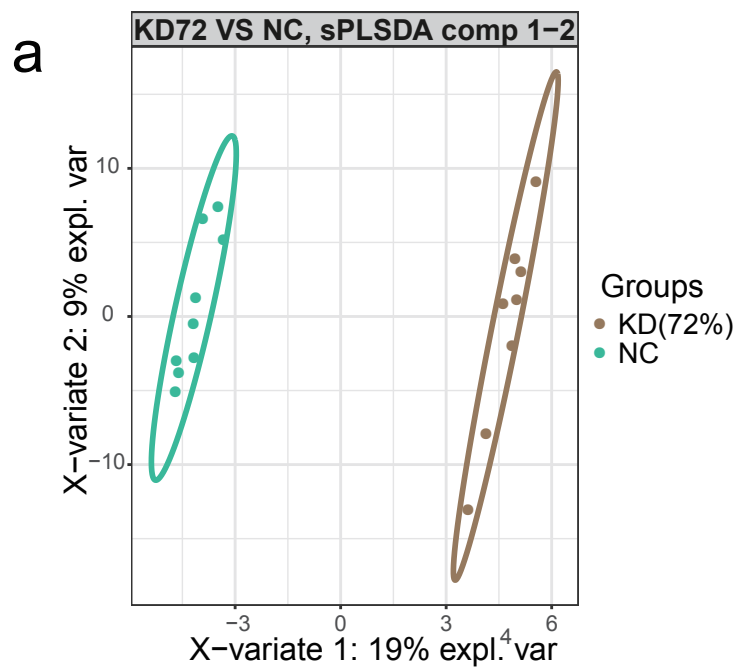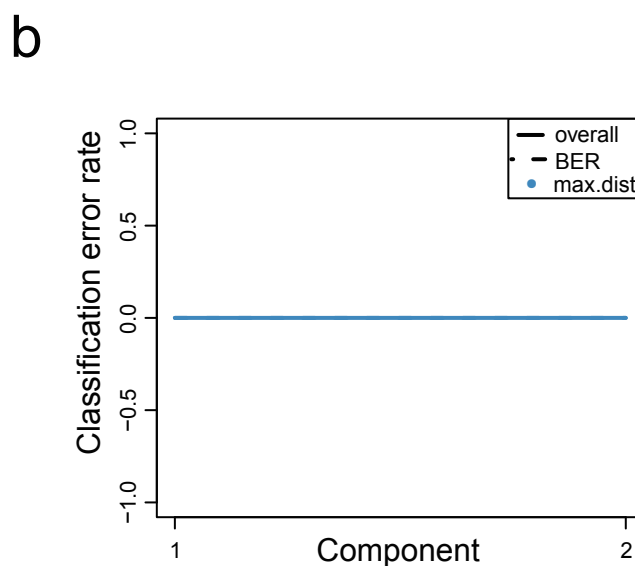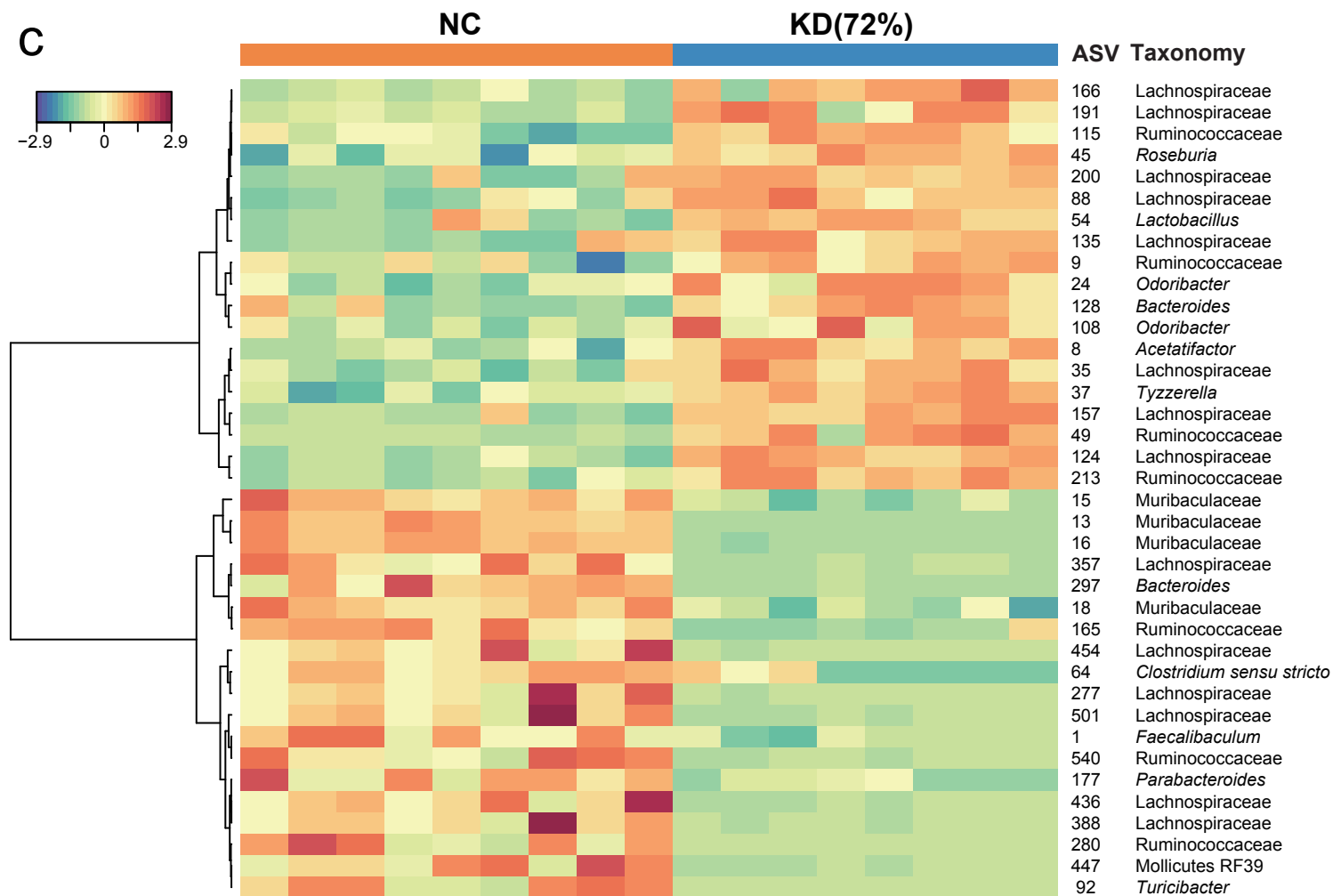

Supplement: FIG S7 [file mBio.03601-20-sf007.pdf]
