# Supplementary material for: Ketogenic Diets Induced Glucose Intolerance and Lipid Accumulation in Mice with Alterations in Gut Microbiota and Metabolites
Source: mBio. 2021 Mar 30;12(2):e03601-20. doi: 10.1128/mBio.03601-20 (PMC8092315; doi:10.1128/mBio.03601-20)
Supplement: TABLE S1 [file mBio.03601-20-st001.docx]

The formulas of diets in animal trial.

| **Research Diets** | | | **customized in FBSH Biotechnology Ltd.** | | | | |
| --- | --- | --- | --- | --- | --- | --- | --- |
| **Ingredients (g)** | **NCR** | **KDR** | **Ingredients (g/kg)** | **NCH** | **KDH** | **KD(89.5%)** | **KD(72%)** |
| Corn Starch | 506.2 | 0 | Corn Starch | 389 | 0 | 0 | 60 |
| Sucrose | 68.8 | 0 | Sucrose | 150 | 0 | 0 | 20 |
| Maltodextrin | 125 | 0 | Maltodextrin | 100 | 0 | 0 | 15 |
| Primex |  | 338 | Vegetable Shortening，hydrogenated | 61.3 | 605 | 562 | 377 |
| Corn Oil |  | 50 | Corn Oil | 8.8 | 86.2 | 86.2 | 76 |
| Soybean Oil | 25 |  |  |  |  |  |  |
| Lard | 20 |  |  |  |  |  |  |
| Casein | 200 | 100 | Casein | 200 | 121 | 165 | 280 |
| DL-Methionine | 3 | 1.5 | DL-Methionine | 3 | 1.56 | 1.56 | 1.56 |
| Cellulose,BW200 | 50 | 50 | Cellulose | 50 | 113 | 113 | 98 |
| Mineral Mix S10001 |  | 35 | Mineral Mix | 13.4 | 23.8 | 23.8 | 23.8 |
| Vitamin Mix V10001C |  | 1 | Vitamin Mix | 10 | 17.8 | 17.8 | 17.8 |
| Mineral Mix S10026 | 10 |  |  |  |  |  |  |
| DiCalcium Phosphate | 13 |  | DiCalcium Phosphate | 7.5 | 24.3 | 24.3 | 24.3 |
| Calcium Carbonate | 5.5 |  | Calcium Carbonate | 6.9 | 4.4 | 4.4 | 4.4 |
| Potassium Citrate, 1H2O | 16.5 |  | Magnesium Oxide | 0.2 | 0.35 | 0.35 | 0.35 |
| Choline bitartrate | 2 | 2 | Choline bitartrate | 0 | 2.5 | 2.5 | 2.5 |
| Carbohydrate (%) | 70 | 0.1 | Carbohydrate (%) | 64.5 | 1 | 0.1 | 8 |
| Protein (%) | 20 | 10.4 | Protein (%) | 20 | 7.7 | 10.4 | 20 |
| Fat (%) | 10 | 89.5 | Fat (%) | 15.5 | 91.3 | 89.5 | 72 |
| kcal/g | 3.85 | 6.76 | kcal/g | 4 | 6.77 | 6.57 | 5.63 |
